# Differential efficacy of olfactory neurospheres from deviated nasal septum and chronic rhinosinusitis patients in regenerating olfactory epithelium

**DOI:** 10.1186/s13287-025-04270-0

**Published:** 2025-04-05

**Authors:** Rong-San Jiang, Chiang-Wen Lee, Yu-Hsuan Lin, Jing-Jie Wang, Jia-Bin Liao, Kuo-Ti Peng, Yao-Chang chiang, Pei-Ling Chi

**Affiliations:** 1 Department of Otolaryngology, Tungs’ Taichung MetroHarbor Hospital, Taichung, Taiwan; 2 Department of Otolaryngology, Taichung Veterans General Hospital, Taichung, Taiwan; 3 Department of Nursing, Division of Basic Medical Sciences, and Chronic Diseases and Health Promotion Research Center, Chang Gung University of Science and Technology, Chiayi, Taiwan; 4 Department of Orthopaedic Surgery, Chang Gung Memorial Hospital, Chiayi, Taiwan; 5 Department of Safety Health and Environmental Engineering, Ming Chi University of Technology, New Taipei City, Taiwan; 6 Department of Otolaryngology, Head and Neck Surgery, Kaohsiung Veterans General Hospital, Kaohsiung, Taiwan; 7 School of Medicine, National Yang Ming Chiao Tung University, Taipei, Taiwan; 8 School of Medicine, Chung Shan Medical University, Taichung, Taiwan; 9 Department of Pathology and Laboratory Medicine, Kaohsiung Veterans General Hospital, Kaohsiung, Taiwan; 10 Graduate Institute of Clinical Medical Sciences, College of Medicine, Chang Gung University, Taoyuan, Taiwan; 11 Department of Medical Education and Research, Kaohsiung Veterans General Hospital, Kaohsiung, 81362 Taiwan; 12 Department of Dental Technology, Shu-Zen Junior College of Medicine and Management, Kaohsiung, Taiwan

**Keywords:** Olfactory neurospheres, Regenerative medicine, Olfactory dysfunction

## Abstract

**Background:**

Olfactory epithelial stem cells hold significant potential for treating olfactory dysfunction by facilitating tissue maintenance and repair. Understanding the inherent qualities of these stem cells is crucial for optimizing their therapeutic efficacy.

**Methods:**

Olfactory epithelial samples were collected from patients with deviated nasal septum (DNS) and chronic rhinosinusitis (CRS). These were cultured to form olfactory neurospheres (ONS), which were then analyzed for neural stem cell markers, neurotrophic factor production, and their ability to differentiate into olfactory sensory neurons (OSNs). The regenerative efficacy of these ONS was tested in a methimazole-induced hyposmic mouse model, with the effects on cellular senescence, apoptosis, and proliferation in the olfactory epithelium assessed.

**Results:**

Both DNS- and CRS-derived ONS exhibited neural stem cell characteristics. DNS-ONS displayed superior self-renewal capacity and higher neurotrophic factor production compared to CRS-ONS, which showed impaired OSN maturation and lower neurotrophic factor levels. In vivo, DNS-ONS were more effective in restoring olfaction, as evidenced by reduced cellular senescence, decreased apoptosis, and increased cell proliferation in the OE of methimazole-induced hyposmic mice.

**Conclusions:**

These findings highlight the importance of selecting the appropriate ONS source for therapeutic applications, with DNS-ONS showing greater promise for olfactory epithelium repair and olfactory function restoration.

**Supplementary Information:**

The online version contains supplementary material available at 10.1186/s13287-025-04270-0.

## Introduction

Loss of smell is a common symptom of olfactory disorders, often caused by head trauma, upper respiratory tract infections, or sinonasal inflammation. These conditions degrade olfactory sensory neurons (OSNs) and damage the olfactory bulb [1, 2]. The abnormal replacement of damaged or senescent OSNs leads to neurogenic exhaustion and olfactory dysfunction [3]. The recent association of COVID-19 with impaired olfactory function [4] underscores the challenges in treating olfactory impairment, especially due to unclear pathogenic mechanisms. Treatments such as olfactory training therapy, intranasal vitamin A, and corticosteroids (both systemic and intranasal) have been reported to improve olfactory function recovery [5], but their therapeutic efficacy remains controversial.

The olfactory epithelium (OE) is a specialized neuroepithelium that includes horizontal basal cells (HBC) and globose basal cells (GBC), which possess neural stem cell properties essential for regenerating new neurons and the entire OE after injury [6]. Numerous studies have demonstrated the benefits of using OE-derived cells to treat various central nervous system (CNS) diseases in both animal models and clinical applications [7–9]. Human OE-derived stem cells were found to ameliorate ipsiversive rotation in hemiparkinsonian rats [10] and increase neovascularization in ischemic mice [11]. These studies indicate that OE-derived stem cells are a promising source for autologous stem cell therapy for patients with olfactory loss.

Growing evidence suggests that certain disease states can alter stem cell properties, resulting in a reduced stem cell population with age, dysregulated proliferation, and diminished functional capacity [12]. Adipose tissues from obese patients have a diminished reservoir of functionally active stem cells, attributed to a reduction in their stemness properties [13]. Progressive cardiovascular disease has been reported, with endothelial progenitor cells (EPCs) undergoing senescent-like changes that impair their regenerative capacity [14]. The efficacy of autologous cell therapy is influenced by how these diseases impact the regenerative potential of stem cells. While stem cell therapy holds promise for treating diseases that are currently incurable with traditional pharmacological methods, many studies have shown that the efficacy of stem cells can be significantly affected by the physiological state of their source. In olfactory disorders, particularly, understanding these stem cell dynamics is crucial for effective therapy. The therapeutic efficacy of OE derived from patients with a differential diagnosis is largely unknown.

Chronic rhinosinusitis (CRS), characterized by persistent inflammation of the sinonasal mucosa, is a prevalent cause of olfactory dysfunction [15]. Prior studies showed chronic olfactory inflammation switched off the olfactory stem cells regenerative phenotype [16], indicating that the function of OE may be impaired in chronic inflammatory rhinosinusitis and OE derived from patients with CRS may lack efficacy in treating disease. This study presents a robust culture model optimized for expanding olfactory neurospheres (ONS) while maintaining their undifferentiated and self-renewable states in vitro. Our objective is to compare the therapeutic effects and differentiation potential of olfactory epithelial-derived cells from patients with deviated nasal septum (DNS) and chronic rhinosinusitis (CRS). By evaluating the differentiation potential and therapeutic efficacy of these cells, this study aims to identify the optimal source of olfactory neurospheres (ONS) for treating patients with anosmia, potentially improving outcomes for those suffering from olfactory dysfunction.

## Materials and methods

### Patients and sample collection

Human OE samples were collected with written informed consent under the approval of the Taichung Veterans General Hospital IRB (CF18350A). Tissue specimens were obtained under general anesthesia during routine rhinosurgical procedures. A single 3–4 mm^2^ biopsy was taken from either the posterior nasal septum or the medial wall of the superior turbinate (see Table 1 for patient details). After collection, tissue specimens were immediately transferred into Hank’s balanced salt solution (HBSS), minced, and digested with collagenase IV for 30 min at 37 °C. The digested tissues were triturated and centrifuged at 300 × g for 5 min. Each piece was then cultured in 6-well plates using ONS medium, consisting of DMEM/F‐12, 1% B‐27 Supplement (Life Technologies Corp.), 10 ng/ml human recombinant basic fibroblast growth factor (bFGF, R&D Systems, Inc., Minneapolis, MN), and 10 ng/ml human recombinant epidermal growth factor (EGF, R&D Systems, Inc.).

**Table 1:** Summary of demographics and surgery types in DNS and CRS patient groups

	DNS (n = 29)	CRS (n = 25)	*P* value
Age, years	31.7 ± 10.34	53.6 ± 12.61	< 0.0001
Gender, male	22 (75.9%)	13 (52.0%)	0.0897
Procedure	Septoplasty	Endoscopic sinus surgery	

Data were showed as mean ± SD or the number (%)

*DNS* Deviated nasal septum, *CRS* Chronic rhinosinusitis

### ONS cultures and differentiation into olfcatory sensory neurons

Two weeks after the initial plating, once confluence was achieved, adherent cells were passaged and cultivated in suspension to form olfactory neurospheres (ONS). The culture media were replaced every 2–3 days to maintain optimal growth conditions. To assess the capability of ONS to differentiate into olfactory sensory neurons (OSNs), ONS were enzymatically treated with Accutase (Innovative Cell Technologies #AT104). The resultant single-cell suspensions were subcultured through six passages in this study. For OSN differentiation, approximately 5 × 10^5^ cells were plated onto Matrigel-coated 6-well plates and cultivated for 4 and 7 days in OSN induction medium, which was supplemented with 300 µM cAMP in ONS medium [17].

### Measurement and image analysis of ONS number and size

To evaluate olfactory neurosphere (ONS) formation, 10^3^ cells were seeded into ultra-low attachment 24-well plates and cultured in ONS medium. Olfactory epithelium (OE) tissue was isolated from patients, and migrated cells were cultured in 24-well plates at a density of 10^3^ cells per well. Non-stem cells adhered to the plates within 1 day, while stem cells formed floating neurospheres. The number of ONS formed was calculated after 1 day, and the diameters of neurospheres were measured after an additional 7 days using phase-contrast photomicrographs. Cell aggregates were classified as neurospheres if they were spherical, optically dense, and had a minimum diameter of 100 µm. Photomicrographs were captured using a Zeiss Axiovert 25 microscope, and sphere diameters were quantified using ImageJ software.

### Immunofluorescence

ONS and differentiated ONS after 7 days were seeded onto Matrigel-coated glass coverslips and cultured overnight. The next day, the spheres were fixed with 4% paraformaldehyde for 15 min at room temperature. Fixed cells were rinsed twice with phosphate-buffered saline (PBS) and blocked with a PBS solution containing 5% bovine serum albumin (BSA) and 0.2% Triton X-100 for 1 h at room temperature. Primary antibodies, diluted in a buffer containing 1% BSA, were incubated with the cells overnight at 4 °C. The next day, secondary antibodies were incubated for 1 h at room temperature. Details regarding primary and secondary antibodies used for immunofluorescence staining can be found in Additional file 1: Table S1. Images of stained cells were captured using a laser scanning confocal microscope (Zeiss LSM 710, Germany) and processed with ImageJ software. In ImageJ, the cell area was demarcated by a 25 µm square region of interest (ROI), and five random ROIs were measured to ascertain the positive area within the OE. A minimum of five fields per coverslip were randomly selected, with the counting spanning three coverslips for each group.

### Flow cytometry

ONS and 7-day differentiated ONS were dissociated with 200 µl of Accutase at 37 °C for 2 min, followed by gentle pipetting to obtain single-cell suspensions. The single-cell suspensions were fixed and stained using BD Cytofix (BD Biosciences, Franklin Lakes, NJ, USA) as per the manufacturer’s instructions. Specific antibodies were used to identify cells in different experimental groups, which were then quantified using an Attune NxT Flow Cytometer (Thermo Fisher Scientific, Waltham, MA, USA).

### Quantitative real-time PCR analysis

Total RNA was extracted using TRIzol (Life Technologies), and cDNA synthesis was performed using the High-Capacity cDNA Reverse Transcription Kit (Applied Biosystems) according to the manufacturer’s instructions. qRT-PCR was performed using the Fast SYBR Green Master Mix (Applied Biosystems) on a StepOnePlus™ System (Applied Biosystems). Primer sequences specific to ONS- and OSN-associated genes are listed in Table S2. For normalization, gene expression levels were referenced to GAPDH.

### Western blot analysis

Cells were lysed using ice-cold RIPA buffer (0.5 M Tris–HCl, 10 mM EDTA, 1.5 M NaCl, 10% NP-40, 2.5% deoxycholic acid, pH 7.4) enriched with a protease inhibitor cocktail (Roche, USA). Protein quantification was conducted using the BCA assay kit (TransGen Biotech, Beijing, China). Equal protein quantities (40 μg) were separated on a 10% SDS-PAGE. Proteins were transferred onto nitrocellulose membranes (PALL, USA) using a transfer buffer (12 mM Tris base, 96 mM glycine, pH 8.3, and 20% methanol). The membranes were blocked for 2 h in TBST buffer containing 5% BSA. Primary antibodies (details in Additional file 1: Table S1) were incubated with the membranes overnight at 4 °C. After three washes with TBST, the membranes were incubated with HRP-conjugated secondary antibodies for 1 h at room temperature. Protein bands were visualized using ECL reagents (Bio-Rad Laboratories, Hercules, CA, USA) and analyzed using ImageLab software (Bio-Rad).

### Nuclear fraction isolation from cells

Nuclear fractions from 7-day differentiated ONS were isolated as previously described [18]. Cells were collected and briefly sonicated for 5 s using a Misonix sonicator XL2020 (Misonix, Inc., USA). The samples were then centrifuged at 8000 × g for 15 min at 4 °C, and the resulting pellet was collected as the nuclear fraction.

### Determination of neurotrophic factors in supernatants

Culture media from ONS and 7-day differentiated ONS were collected and centrifuged at 13,000 × g for 15 min to remove cell debris. Neurotrophic factor levels in the supernatants were quantified using Luminex X-MAP technology with the Human Premixed Multi-Analyte kit (R&D Systems, Wiesbaden, Germany).

### Mouse model of hyposmia and ONS treatment

Four-week-old male NOD/SCID mice (18–20 g) were obtained from the National Laboratory Animal Center, Taiwan. Male mice were selected to minimize hormonal variability, which could otherwise affect the consistency of the results [19]. Five animals were housed per cage in individually ventilated cages (IVCs). The animal vivarium was a specific pathogen-free (SPF) facility with temperature and humidity control (21 ± 3 °C, 50 ± 10%). Animals were maintained under a reversed light–dark cycle (lights off 09:00 AM–09:00 PM). All experimental procedures complied with European ethical regulations (Directive 2010/63/EU) and were approved by the Institutional Animal Care and Use Committee (IACUC) of Kaohsiung Veterans General Hospital, Taiwan (Ref. 2019-2022-A038). This study was conducted and reported in accordance with the ARRIVE guidelines 2.0 [20].

For cell transplantation, neurospheres were dissociated into single cells at a 1:3 split ratio. Two portions were cryopreserved in liquid nitrogen, each containing the number of cells required for two injections, while one portion was cultured and stored for future use. Prior to injection, cells were thawed, resuspended in PBS, and cell viability was assessed. Approximately 5 × 10^6^ viable cells were injected in 40 µl of PBS. Cell viability and injection details are provided in Table 2.

**Table 2:** Summary of donor characteristics, injection concentrations, and post-freeze viability for DNS-ONS and CRS-ONS

Donor category	Age (years)	Gender	1st Injection (cells/mice)	2nd Injection (cells/mice)	3rd Injection (cells/mice)	4th Injection (cells/mice)	Cell concentration (pre-freeze, cells/ml)	Cell concentration (post-freeze, cells/ml)	Viability (%)
DNS-ONS	27	F	5.1 × 10^6^	4.8 × 10^6^	4.5 × 10^6^	5.3 × 10^6^	3.0 × 10^7^	1.7 × 10^7^	56
	38	M	4.5 × 10^6^	4.5 × 10^6^	5.7 × 10^6^	5.0 × 10^6^	4.4 × 10^7^	3.6 × 10^7^	82
	29	M	4.8 × 10^6^	5.0 × 10^6^	4.5 × 10^6^	5.8 × 10^6^	4.5 × 10^7^	4.0 × 10^7^	89
	31	F	4.8 × 10^6^	5.1 × 10^6^	5.3 × 10^6^	5.5 × 10^6^	4.1 × 10^7^	4.1 × 10^7^	99
	36	F	5.4 × 10^6^	5.4 × 10^6^	4.6 × 10^6^	5.1 × 10^6^	4.6 × 10^7^	4.0 × 10^7^	87
CRS-ONS	72	M	4.8 × 10^6^	5.0 × 10^6^	4.5 × 10^6^	5.0 × 10^6^	4.2 × 10^7^	4.1 × 10^7^	98
	61	M	4.5 × 10^6^	4.7 × 10^6^	5.6 × 10^6^	5.0 × 10^6^	6.6 × 10^7^	4.8 × 10^7^	73
	55	F	5.0 × 10^6^	5.6 × 10^6^	5.0 × 10^6^	5.6 × 10^6^	6.6 × 10^7^	4.2 × 10^7^	63
	49	M	5.6 × 10^6^	4.6 × 10^6^	5.0 × 10^6^	5.6 × 10^6^	9.6 × 10^7^	5.2 × 10^7^	54
	64	M	5.3 × 10^6^	5.5 × 10^6^	5.6 × 10^6^	4.7 × 10^6^	4.5 × 10^7^	4.3 × 10^7^	90

A schematic of the experimental design is shown in Fig. 5A. Olfactory nerve degeneration was induced by intraperitoneal injection of methimazole (MI) at a dose of 75 mg/kg (#301507, Sigma), administered weekly for 2 weeks, as previously described [21, 22]. After MI treatment, mice were randomly assigned to one of three groups (n = 5 per group): (1) saline (vehicle control), (2) DNS-ONS, or (3) CRS-ONS. Treatments began 2 weeks post-MI injection. DNS-ONS or CRS-ONS was administered intranasally (i.n.) to anesthetized mice (using ketamine, 80 mg/kg, and xylazine, 10 mg/kg). Each mouse received 20 µl of treatment in one nostril, followed by a 3–5 min absorption period before administering 20 µl to the other nostril, once a week for 4 weeks. Following treatment, mice were euthanized via intraperitoneal injection of ketamine (200 mg/kg) and xylazine (20 mg/kg). Olfactory epithelium tissues from all 20 mice were collected for subsequent analysis.

### Biodistribution of PKH26-labelled DNS-ONS

PKH26 dye (PKH26GL, Sigma-Aldrich Co.) was diluted to a final concentration of 15 µM in 100 µL of diluent C. Either 5 × 10^6^ cells or PBS (as control) were incubated with the dye at room temperature for 15 min. Following incubation, the cells were washed with PBS and centrifuged at 300 × g for 5 min. The resulting pellet was resuspended in 40 µL of PBS. These PKH26-labelled cells or PKH26-PBS control were administered intranasally to MI-treated mice 24 h before tissue harvesting. Imaging was performed using the IVIS Spectrum (Xenogen, Perkin Elmer) with excitation and emission wavelengths set to 570 nm and 595 nm, respectively. Data analysis was conducted using Living Image Software for IVIS.

### Behavioral tests

To assess olfactory function, the food-finding test was employed. Mice were evaluated weekly for 3 weeks prior to MI injection and weekly post-injection until euthanasia. For validation of the hyposmia mouse model, mice were food-deprived for 48 h before being placed in a T-maze. A cheese pellet was hidden beneath sawdust in one of the two arms of the maze, with its location randomized for each trial. The time taken for the mice to locate the food was recorded. Control mice typically found the cheese within 40 s, whereas MI-treated mice consistently failed to locate it within the 3-min timeframe.

### Histological and immunohistochemical examination

After perfusion with saline and 4% paraformaldehyde, the head samples were fixed in 4% paraformaldehyde overnight at 4 °C. The samples were then decalcified using Decalcifier II (Leica Biosystems) for 2 days. Subsequently, the samples were sequentially immersed in 10% sucrose for 1 day and 20% sucrose the following day, before being embedded in paraffin. Sections, 10 µm-thick, were stained with hematoxylin and eosin (H&E) to evaluate the OE thickness. Immunohistochemistry was performed using markers specified in Table S1. Images were captured using a Zeiss fluorescence microscope.

### Statistical analysis

Statistical analyses were performed on data from animal studies and cellular experiments, presented as the mean ± standard deviation (SD) from at least four independent trials. Data were analyzed using GraphPad Prism 9 (GraphPad Software, La Jolla, CA, USA). Comparisons involving three or more groups were evaluated using one-way or two-way ANOVA, followed by Tukey’s post hoc test. A *P* value of less than 0.05 was considered statistically significant.

## Results

### Neurosphere generation from DNS and CRS patient olfactory epithelium

**Fig. 1:**
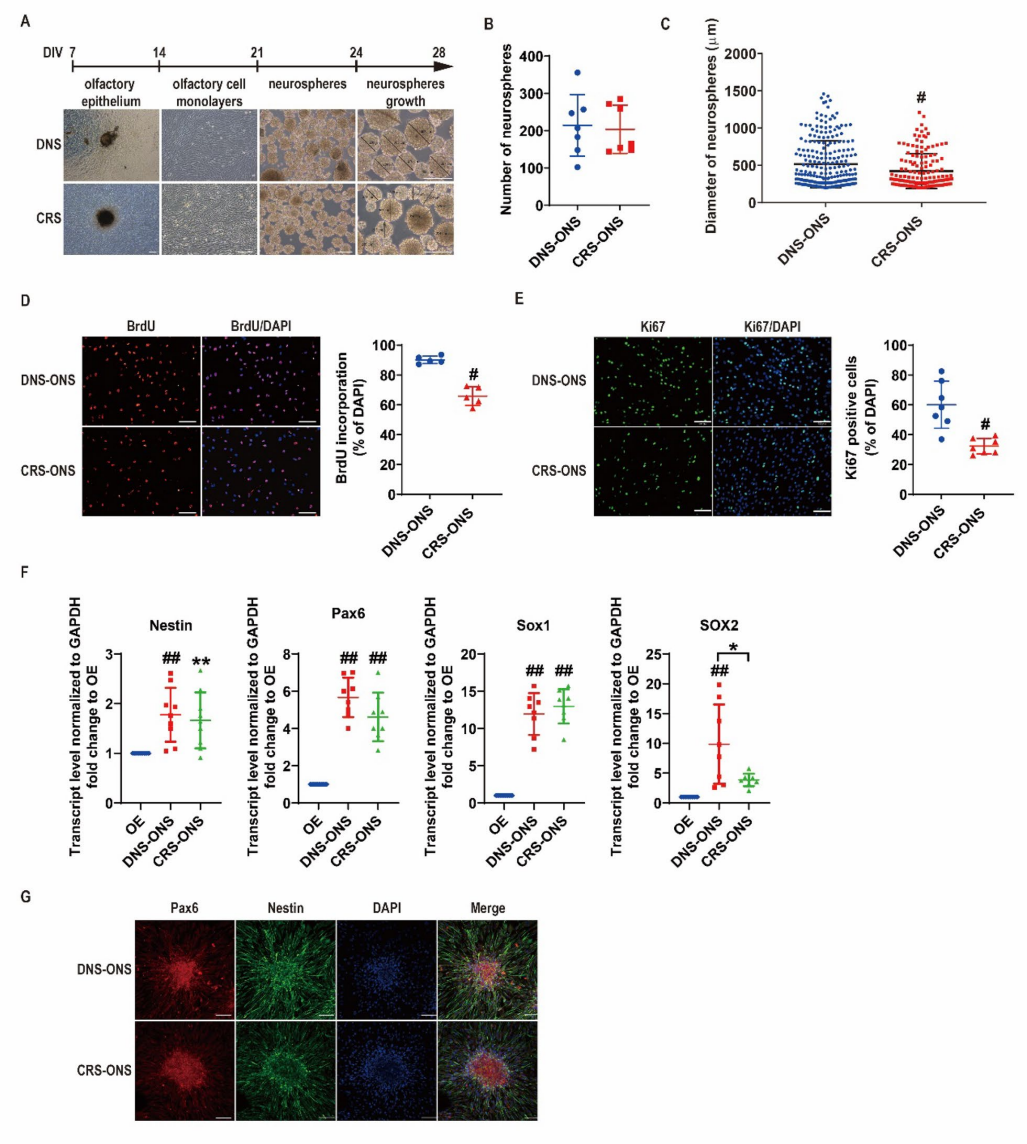
Generation of olfactory epithelium-derived ONS from patients with DNS and CRS. **A** Phase-contrast images illustrating the generation process of ONS from the olfactory epithelium (OE) of DNS and CRS patients, with a schematic flow diagram. Scale bars, 200 µm. **B** Quantification of neurosphere numbers one day after seeding cells. **C** Comparison of ONS diameters (≥ 250 µm) measured at 28 days post-culture between DNS and CRS patients. **D** Proliferation assessment using BrdU staining. Nuclei were counterstained with DAPI (blue), and dividing cells were labeled with anti-BrdU antibody (red). The percentage of BrdU-positive cells is quantified. Scale bars, 100 µm. **E** Immunostaining of ONS from DNS and CRS patients for the proliferation marker Ki67. Scale bars, 100 µm. Percentage of Ki67-positive cells, calculated by the ratio of green-stained cells to blue DAPI-stained nuclei. **F** Relative gene expression of neural stem cell (NSC) markers Nestin, Pax6, SOX1 and SOX2 in DNS-ONS and CRS-ONS at day 21, compared to OE, measured by RT-PCR. **G** Immunostaining of DNS- and CRS-derived ONS for NSC markers Pax6 and Nestin. Scale bars, 100 µm. Statistical analysis was performed using one-way ANOVA with Tukey’s post-hoc test. Data values are represented as mean ± standard deviation. #*P* < 0.01 comparing DNS-ONS to CRS-ONS; **P < 0.05, ##*P* < 0.01 comparing OE to ONS (n = 7 per group)

The long-term genetic stability of human OE cell cultures is influenced by factors such as age, sex, donor source, and in vitro manipulation, all of which impact their potential clinical applications [23]. Although protocols for optimizing sphere cultures from OE-derived cells are well-established, successfully generating spheres from all patient biopsies remains challenging. Biopsy specimens were collected from the lateral nasal septum, medial to the superior turbinate, of patients with deviated nasal septum (DNS) and chronic rhinosinusitis (CRS). Table 1 summarizes the details of the 54 specimens, comprising 29 from DNS patients and 25 from CRS patients. OE tissues were isolated, and the migrated cells were cultured on 24-well plates at a seeding density of 103 cells per well. After 1 day, the number of neurospheres formed was comparable between DNS-derived ONS (DNS-ONS) and CRS-derived ONS (CRS-ONS) (Fig. 1B). However, after 7 days in culture, DNS-ONS neurospheres exhibited significantly larger diameters than CRS-ONS neurospheres (Fig. 1C), indicating more robust growth in DNS-ONS. Further analyses revealed that DNS-ONS showed higher BrdU incorporation and Ki67 expression compared to CRS-ONS (Fig. 1D, E), suggesting increased proliferative activity and a greater capacity to enter the S-phase. RNA analysis demonstrated that both DNS-ONS and CRS-ONS expressed neural stemness markers (Nestin, Pax6, SOX1, and SOX2) at significantly higher levels than OE tissue (Fig. 1F). Notably, SOX2 mRNA levels were significantly lower in CRS-ONS than in DNS-ONS. Immunostaining confirmed these findings, highlighting distinct Nestin and PAX6 expression patterns within the ONS (Fig. 1F). Although CRS-ONS exhibited reduced proliferation compared to DNS-ONS, both types could be successfully isolated and expanded, demonstrating similar neural stem cell properties under the applied culture conditions.

### OE-derived ONS express HBC and GBC markers

**Fig. 2:**
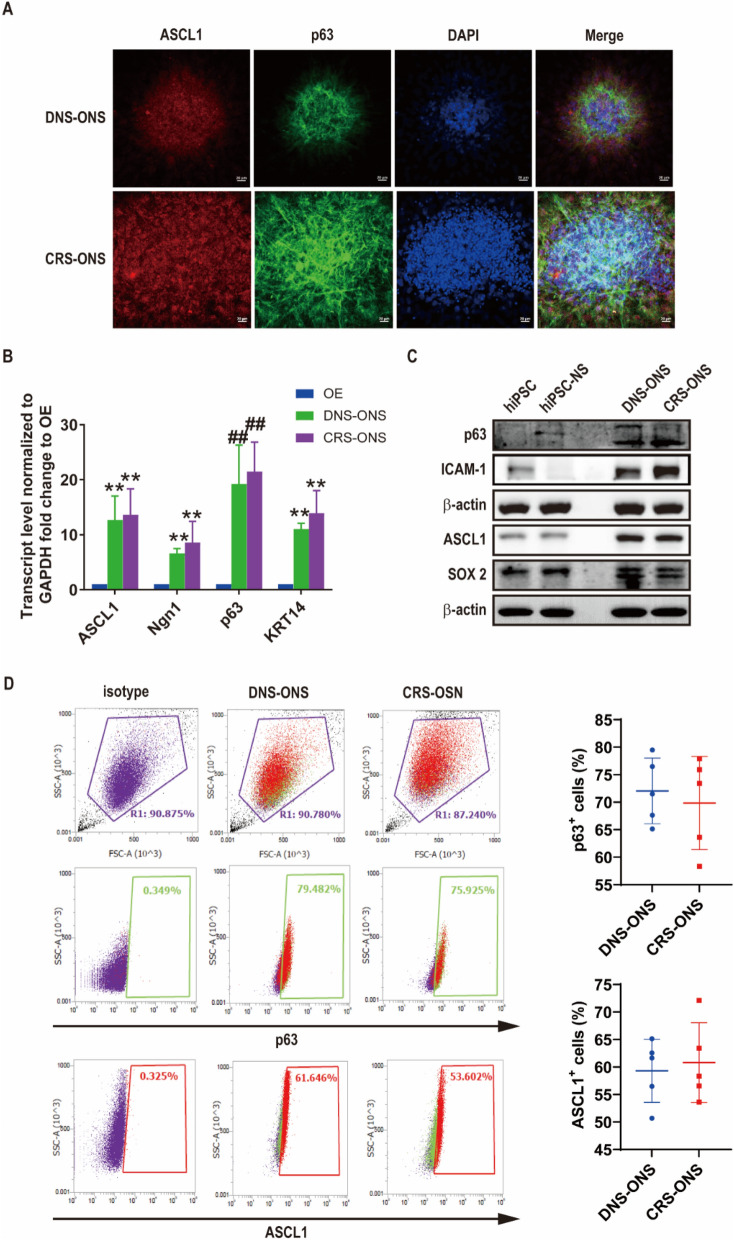
Markers for HBC and GBC in ONS derived from human OE. **A** Immunostaining images showing ONS derived from DNS and CRS patients, stained for HBC marker p63 and GBC marker ASCL1. Scale bars, 20 µm. **B** Relative gene expression of HBC markers (p63 and KRT14) and GBC markers (ASCL1 and Ngn1) in DNS- and CRS-ONS at day 21, compared to OE, quantified by RT-PCR. **C** Western blot analysis of HBC markers (p63 and ICAM-1) and GBC markers (ASCL1 and SOX-2) in hiPSCs, hiPSC-neurospheres, DNS-ONS, and CRS-ONS. **D** Flow cytometry analysis of intracellular levels of p63 and ASCL1 in DNS- and CRS-ONS. Statistical analyses were performed using two-way ANOVA with Tukey’s post-hoc test. Data are presented as mean ± standard deviation. **P < 0.05 and ##*P* < 0.01 comparing OE to ONS (n = 5 per group)

The OE’s capacity for neurogenesis is due to HBC and GBC, characterized by self-renewal, multipotency, and quiescence [24]. We investigated HBC and GBC populations in ONS from DNS and CRS patients using immunostaining and real-time PCR. Immunostaining showed that ONS from both patient types were positive for p63 (HBC marker) and ASCL1 (GBC marker) (Fig. 2A). Real-time RT-PCR confirmed the mRNA expression of GBC (ASCL1, Ngn1) and HBC (p63, KRT14) markers in neurospheres compared to OE (Fig. 2B). Consistent with previous findings [25, 26], we observed that OE-derived neurospheres included heterogeneous populations of both HBC and GBC. Western blot analysis confirmed GBC and HBC marker expression in OE-derived neurospheres, while hiPSCs and hiPSC-derived neurospheres did not express these markers (Fig. 2C). SOX2 expression was detected in hiPSCs, hiPSC-NS, and ONS. Flow cytometry revealed higher percentages of p63- and ASCL1-positive cells in DNS-ONS (72.05 ± 5.96% and 59.31 ± 5.76%) compared to CRS-ONS (69.84 ± 8.46% and 60.81 ± 7.26%) (Fig. 2D), with no significant differences in ASCL1 and p63 expression among the groups. These findings suggest that ONS from DNS and CRS patients have the potential to develop into olfactory stem cells, marked by both GBC and HBC populations, which is not observed in hiPSC-NS.

### Differentiation of olfactory neurospheres into sensory neurons

**Fig. 3:**
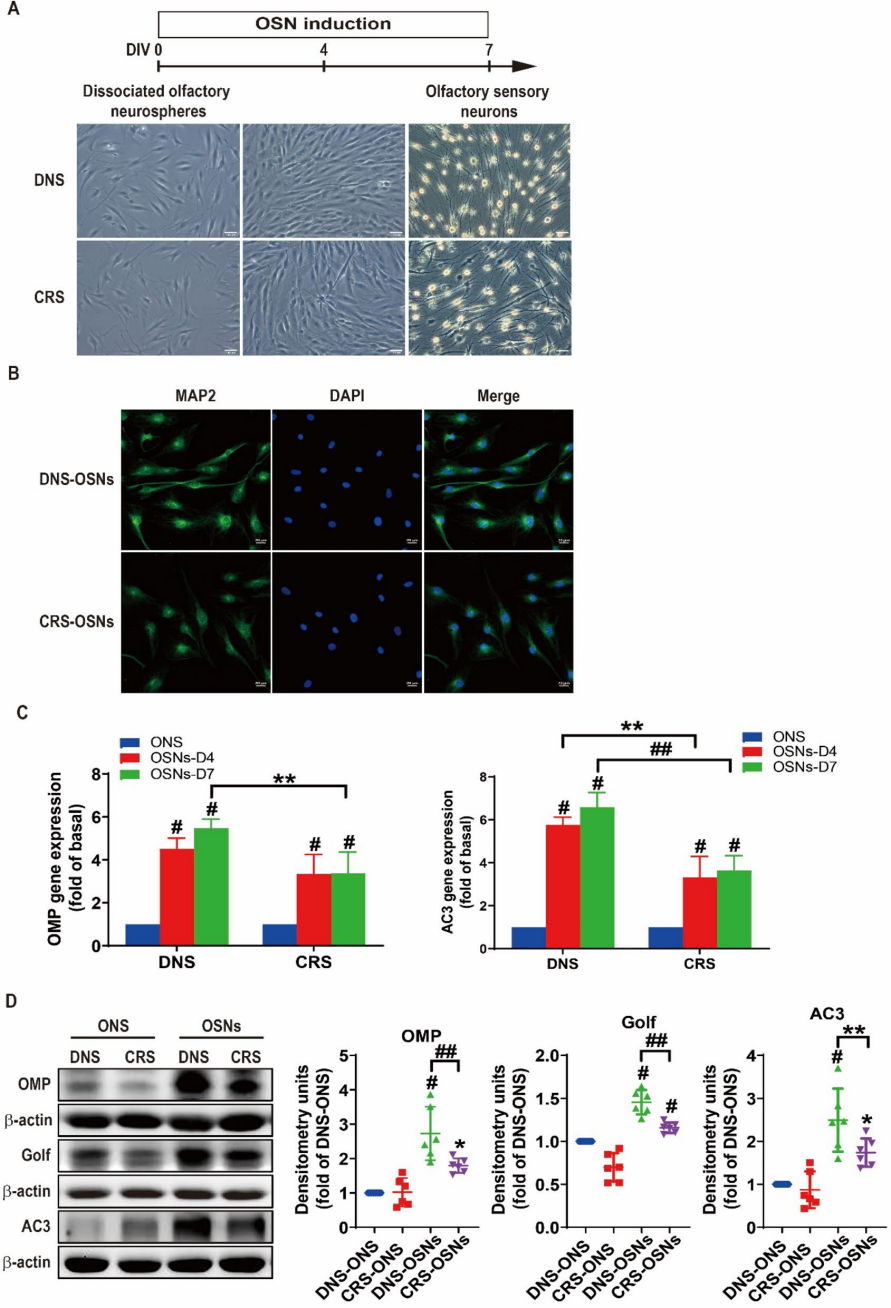

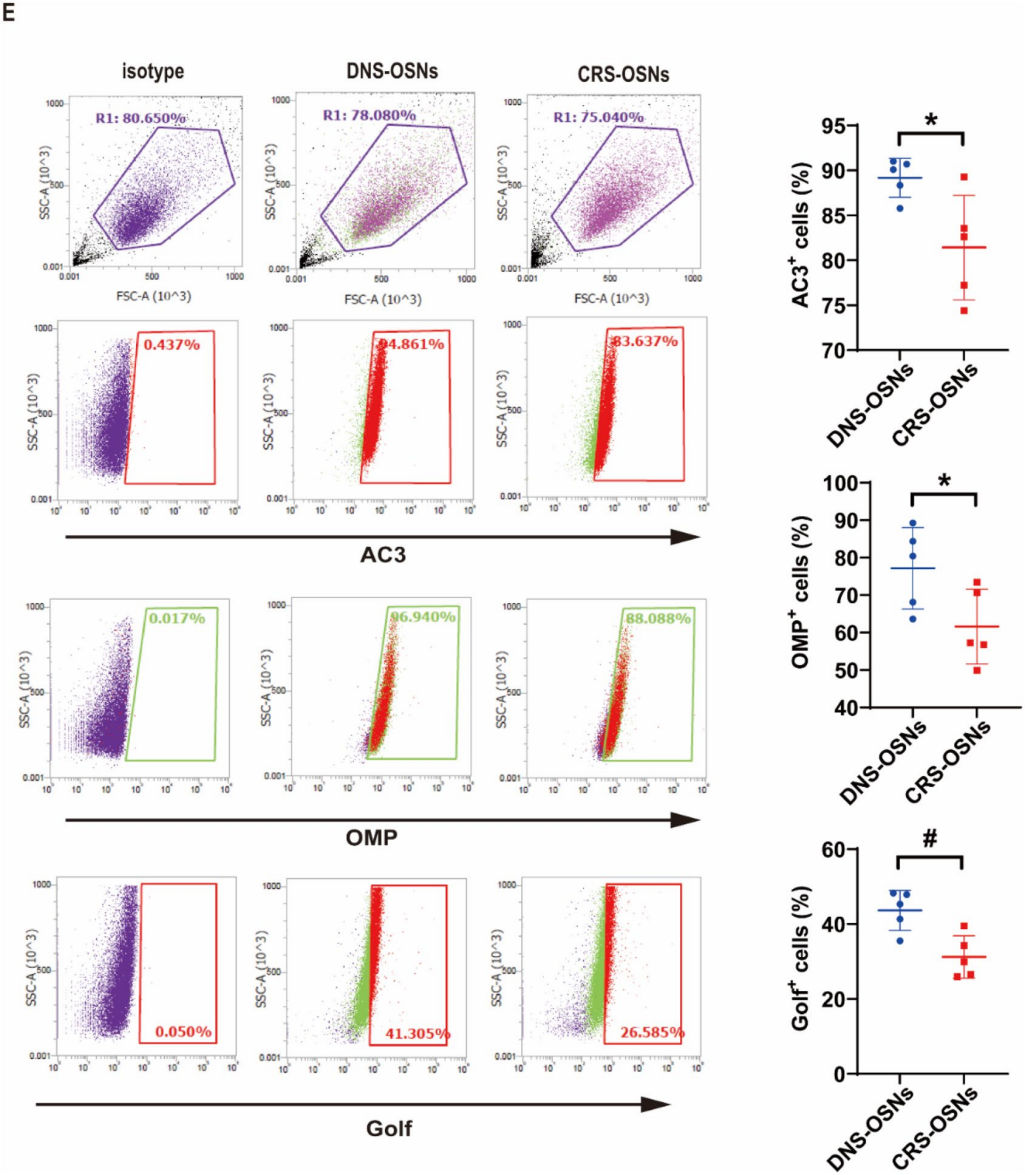
ONS differentiation into OSNs. **A** Phase-contrast images depicting the differentiation of OSNs from DNS- and CRS-ONS, supplemented by a schematic flow diagram. Scale bars, 100 µm. **B** Immunostaining images of OSNs from DNS and CRS patients at day 7 post-OSN induction, stained for MAP2 and DAPI. Scale bars, 20 µm. **C** Relative gene expression levels of OSN markers (OMP and AC3) in DNS- and CRS-ONS subjected to OSN induction medium at days 4 and 7, compared to undifferentiated cells, measured using RT-PCR. **D** Western blot analyses showing expression patterns of OSN markers (OMP, Golf, and AC3) in DNS- and CRS-ONS, as well as DNS- and CRS-OSNs at day 7 post-OSN induction. **E** Flow cytometric evaluation of OSN markers—OMP, Golf, and AC3—in DNS- and CRS-derived OSNs. Statistical evaluations were performed using two-way ANOVA for part (**B**), and one-way ANOVA for parts (**D**, **E**), followed by Tukey’s post-hoc test. Data are presented as mean ± standard deviation. *P < 0.05 and #*P* < 0.01 for differentiating ONS from OSNs groups; **P < 0.05 and ##*P* < 0.01 comparing DNS- to CRS-OSNs (n = 5 per group)

To assess the regenerative capacity of ONS in OE neurogenesis, neurospheres were dissociated into single-cell suspensions and incubated with OSN induction medium for 4 and 7 days. The morphology of ONS single cells at 4 and 7 days post-induction is shown in Fig. 3A. MAP2 immunocytochemistry was performed to assess the maturation and functionality of OSNs derived from DNS-ONS and CRS-ONS. As shown in Fig. 3B, cells derived from both DNS-ONS and CRS-ONS expressed MAP2, a marker of mature neurons. Differentiated cells from DNS-ONS exhibited a more organized cytoskeletal structure, characterized by microtubules extending along actin filaments, compared to the less organized structure observed in CRS-ONS-derived cells. This suggests that DNS-ONS have a greater capacity for neuronal differentiation and cytoskeletal organization, indicative of enhanced maturation and functionality compared to CRS-ONS-derived neurons. Gene expression of olfactory sensory neuronal proteins in DNS- and CRS-ONS-derived cells was assessed on days 4 and 7 using real-time PCR. The levels of olfactory marker protein (OMP) and adenylyl cyclase 3 (AC3) increased over time during OSN differentiation. Notably, DNS-ONS differentiated cells exhibited higher OMP and AC3 expression than CRS-ONS cells (Fig. 3C). Western blot analysis confirmed that DNS-ONS differentiated cells had higher levels of OMP, Golf, and AC3 than CRS-ONS cells on day 7 post-induction (Fig. 3D). Flow cytometry revealed higher percentages of AC3, OMP, and Golf expression in DNS-ONS differentiated cells (77.17 ± 10.87%, 89.18 ± 2.16%, 43.67 ± 5.32%) compared to CRS-ONS cells (61.61 ± 9.98%, 81.42 ± 5.80%, 31.23 ± 5.67%) (Fig. 3E). These results demonstrate that ONS from DNS and CRS patients can differentiate into OSNs, with DNS-ONS achieving more complete maturation compared to CRS-ONS.

### Olfactory neuron maturation deficits in CRS patients

Immature olfactory neurons express growth-associated protein-43 (GAP-43) and TrkB [27, 28]. OMP expression is localized in the nucleus of mature neurons, while immature neurons show reduced OMP levels predominantly in the cytoplasm [27]. We analyzed GAP-43, TrkB, and OMP localization to validate whether CRS-derived ONS exhibit defects in OSN maturation.

**Fig. 4:**
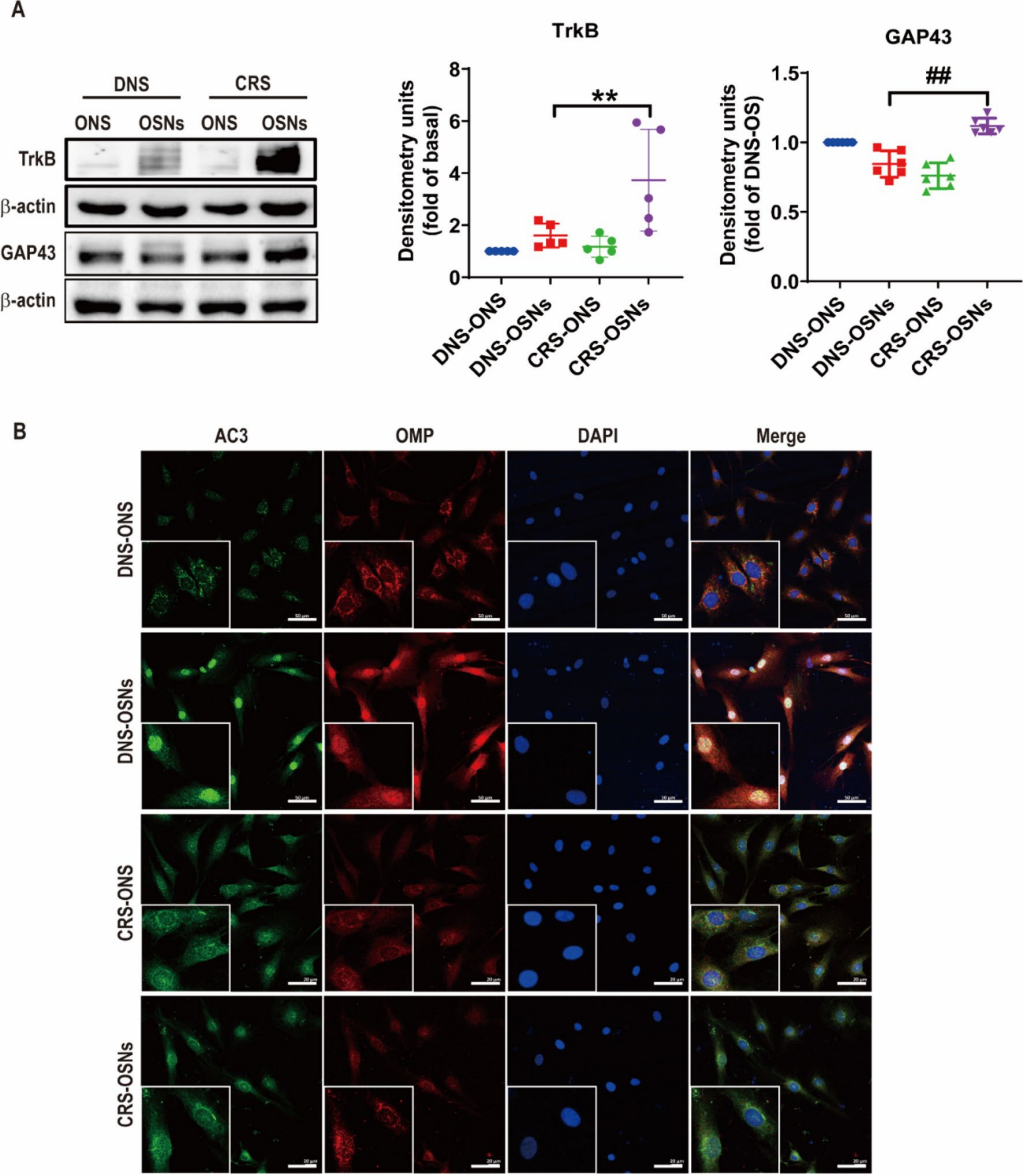

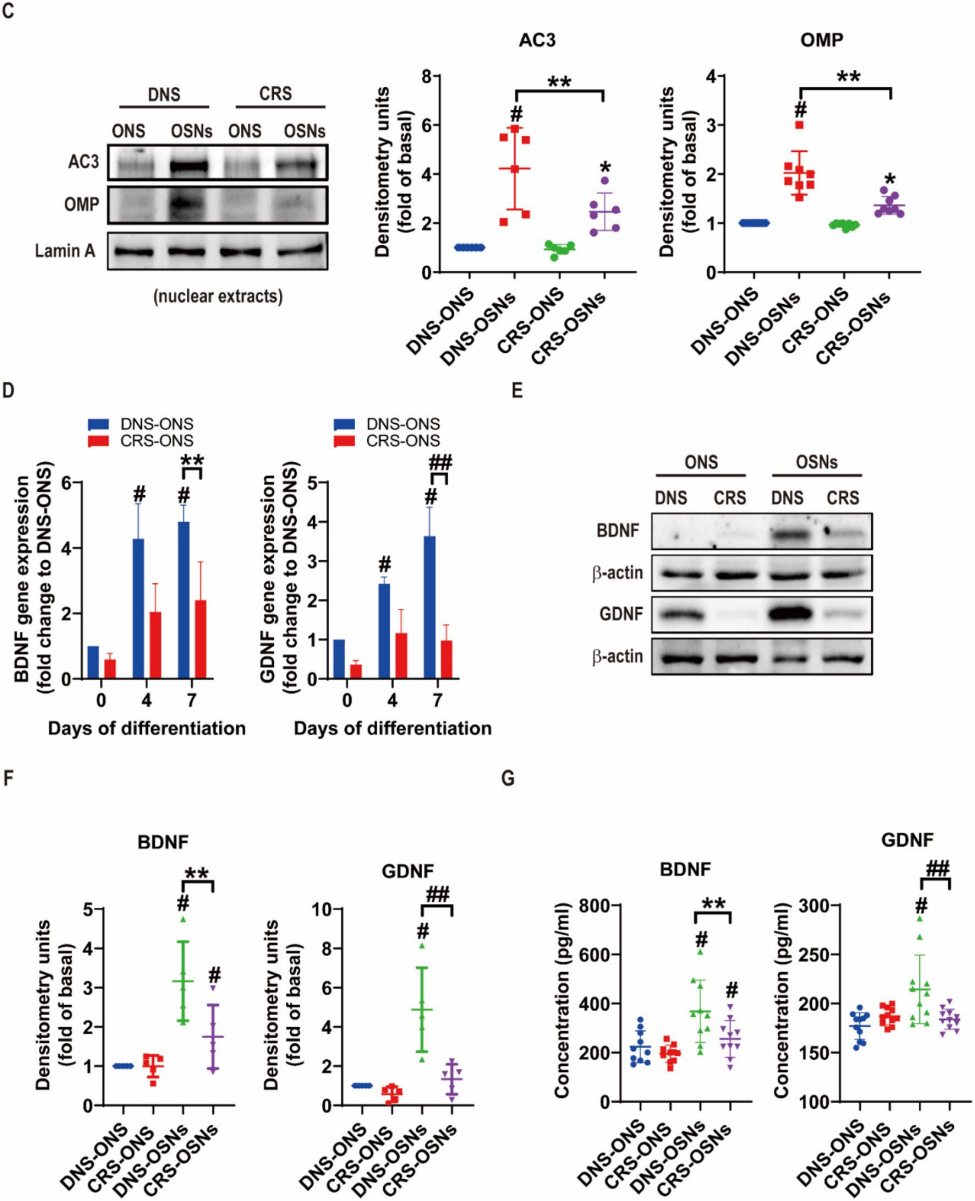
Impaired differentiation of CRS-ONS into OSNs. **A** Western blot analysis of immature OSN markers TrkB and GAP43 in DNS- and CRS-ONS, and DNS- and CRS-OSNs at day 7 post-OSN induction. **B** Immunostaining images of ONS and OSNs from DNS and CRS patients at day 7, stained for OMP and AC3. Scale bars, 20 µm. **C** Western blot analysis of nuclear fractions from DNS- and CRS-ONS and OSNs collected at day 7 post-induction, with Anti-LaminA as a control. **D** RT-PCR quantification of relative gene expression levels of BDNF and GDNF in DNS- and CRS-ONS exposed to OSN induction medium on days 4 and 7, compared to undifferentiated cells. **E** Western blot analysis of BDNF and GDNF expression in DNS- and CRS-ONS and DNS- and CRS-OSNs at day 7 post-induction. **F** Luminex assays quantifying BDNF and GDNF levels in the culture supernatants of DNS- and CRS-ONS, untreated and after OSN induction medium treatment on day 7 (n = 11 per group). Statistical analyses used one-way ANOVA with Tukey’s post-hoc test. Data are presented as mean ± standard deviation. *P < 0.05 and #*P* < 0.01 differentiate ONS from OSNs groups; **P < 0.05 and ##*P* < 0.01 comparing DNS- to CRS-OSNs (n = 5 per group)

CRS-ONS-derived OSNs exhibited higher expressions of GAP-43 and TrkB compared to DNS-ONS-derived OSNs (Fig. 4A). Immunostaining and nuclear fraction analyses showed AC3 and OMP in the nucleus of DNS-OSNs but in the cytosol of CRS-OSNs and undifferentiated cells (Fig. 4B, C). Both BDNF and GDNF, known to be released from the OE, enhance OSN survival [29–31]. Real-time PCR showed a significant increase in BDNF and GDNF levels during DNS-OSN differentiation, with a slight increase in CRS-OSN differentiation. Moreover, on the 7th day following OSN induction, DNS-ONS differentiated cells showed greater levels of BDNF and GDNF compared to CRS-ONS differentiated cells (Fig. 4D). Western blot showed higher BDNF and GDNF levels in DNS-ONS than in CRS-ONS differentiated cells (Fig. 4E, F). BDNF secretion was higher in DNS-ONS than in CRS-ONS differentiated cells. While DNS-ONS showed increased GDNF secretion compared to undifferentiated cells, CRS-ONS did not show a significant difference (Fig. 4G).

### Efficacy of DNS and CRS-derived ONS in hyposmic mice

**Fig. 5:**
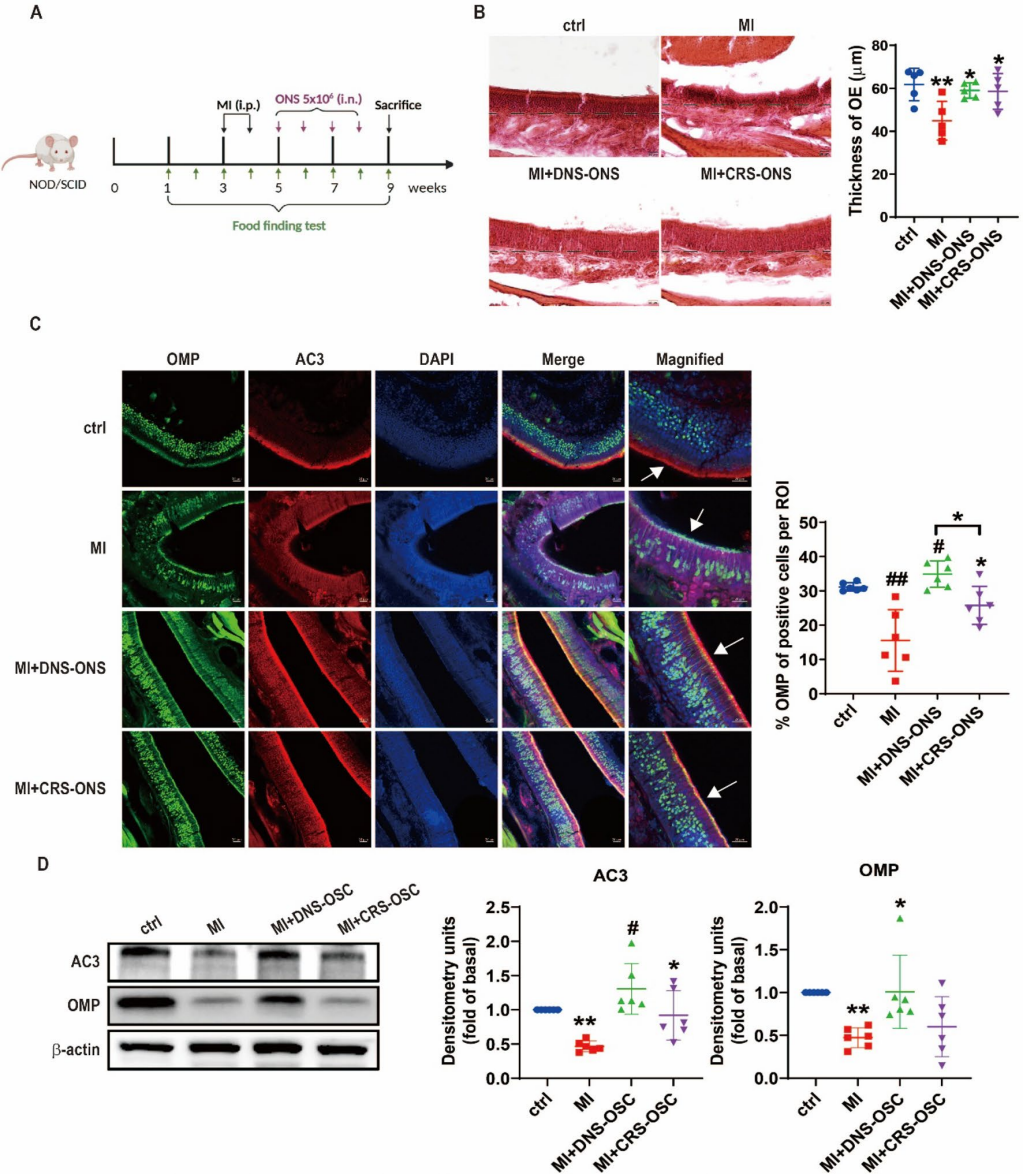

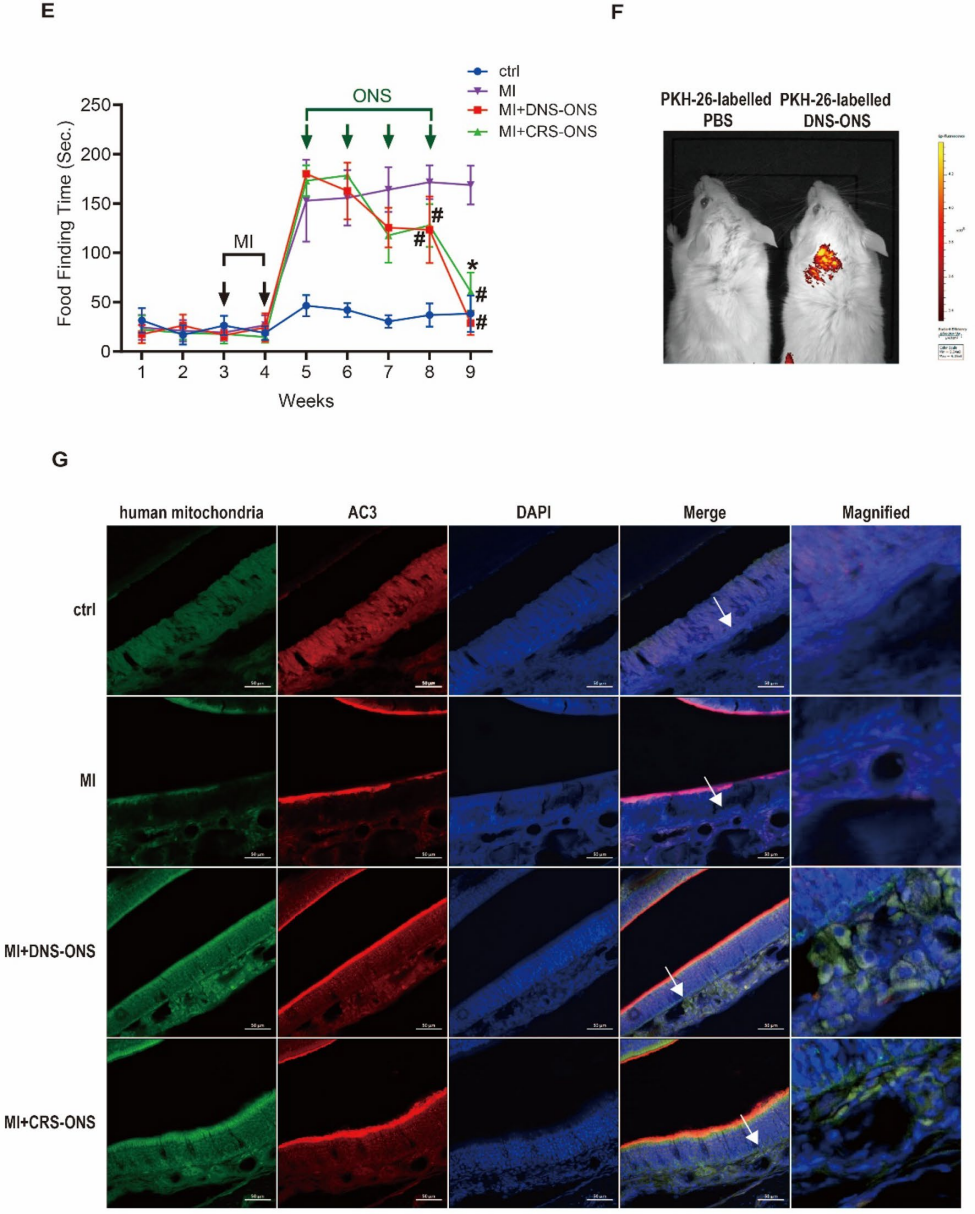
Impact of DNS- and CRS-ONS on olfactory function recovery in a MI-induced hyposmic mouse model. **A** Timeline and schematic of the MI-induced hyposmic mouse model, illustrating the treatment regimen with DNS- and CRS-ONS. **B** Hematoxylin and eosin-stained sections of the olfactory epithelium (OE) from experimental mice. The OE thickness was measured using ImageJ software. Scale bar, 20 µm. **C** Immunofluorescence images showing the expression of olfactory sensory neuron (OSN) markers OMP and AC3 in OE sections, with DAPI used for nuclear counterstaining. Quantitative analysis of OMP in OE sections was performed with three samples per group, and two images per sample. Scale bars: 20 µm. **D** Western blot analysis of AC3 and OMP protein levels in OE tissues. **E** Quantitative analysis of the time taken by mice to locate food in the food-finding test. **F** Representative IVIS images taken 24 h after intranasal administration of PKH-26-labelled PBS or DNS-ONS to mice. **G** Immunofluorescence images showing the expression of the human-specific mitochondrial antibody (hMit) and AC3 in OE sections, with DAPI used for nuclear counterstaining. Statistical analyses were performed using one-way ANOVA with Tukey’s post-hoc test. Data are presented as mean ± standard deviation. *P < 0.05 and #*P* < 0.01 for comparisons between MI and either DNS- or CRS-ONS treatment groups; **P < 0.05 and ##*P* < 0.01 for comparisons between control and MI groups (n = 5 per group)

We used a methimazole (MI)-induced NOD/SCID mouse model [21] to investigate the impact of DNS- and CRS-derived olfactory neurospheres (ONS) on olfactory neuroepithelium. Mice received intranasal administrations of DNS- or CRS-ONS for 4 weeks (Fig. 5A). H&E staining revealed that MI-induced mice had atrophied olfactory neurons and disrupted neuronal arrangement. However, after DNS- and CRS-ONS administration, there was a remarkable recovery in the structural integrity of these neurons (Fig. 5B). Immunofluorescence studies showed that OMP expression, compromised in MI-induced mice, was restored in DNS- and CRS-ONS treated mice. AC3-positive cells, scarcely detectable in MI-induced mice, were mainly located in the apical OE regions in DNS- and CRS-ONS treated groups (Fig. 5C). Protein levels of AC3 and OMP were also higher in DNS-ONS treated mice (Fig. 5D). This was mirrored at the protein level for both AC3 and OMP in the OE, especially with DNS-OSC treatment in MI-induced mice (Fig. 5D). Before MI induction, mice found the food pellet in 20 ± 9 s. After a second MI induction, this increased to 168 ± 26 s. Following 8 weeks of DNS- or CRS-ONS treatment, the time decreased to 123 ± 30 and 127 ± 19 s, respectively. By the 9th week, DNS-treated mice found the food in 28 ± 12 s, while CRS-ONS treated mice took 60 ± 19 s (Fig. 5E). Fluorescence imaging confirmed a detectable signal in the brain 24 h after the final intraperitoneal injection of PKH-26-labeled DNS-ONS or PKH-26-labeled PBS (Fig. 5F). Additionally, we performed double immunofluorescence staining on sections of the mouse olfactory epithelium using a human-specific mitochondria antibody (hMit) and ADCY3, a marker for olfactory sensory neurons reactive across human, mouse, and rat species. This analysis distinguished endogenous cells from transplanted human cells. As shown in Fig. 5G, hMit-positive cells were identified in the olfactory epithelium, confirming the presence of transplanted human cells. These results demonstrate that the efficacy of ONS in treating hyposmia depends on its source. DNS-derived ONS consistently exhibited superior restorative effects in terms of protein expression, neuronal recovery, and functional outcomes compared to CRS-derived ONS.

### DNS-ONS reduces cellular senescence, inhibits apoptosis, and enhances proliferation in MI-induced mice

**Fig. 6:**
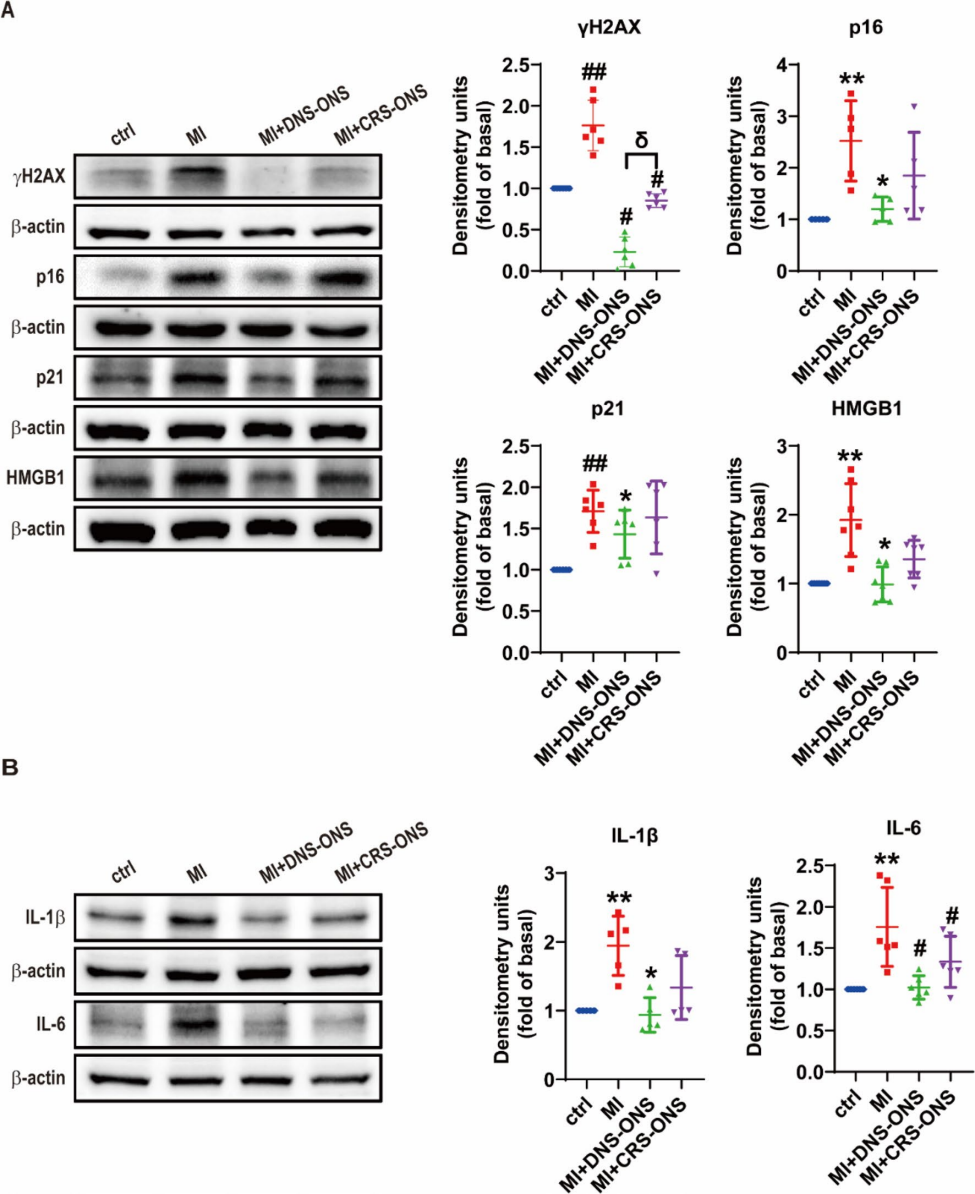

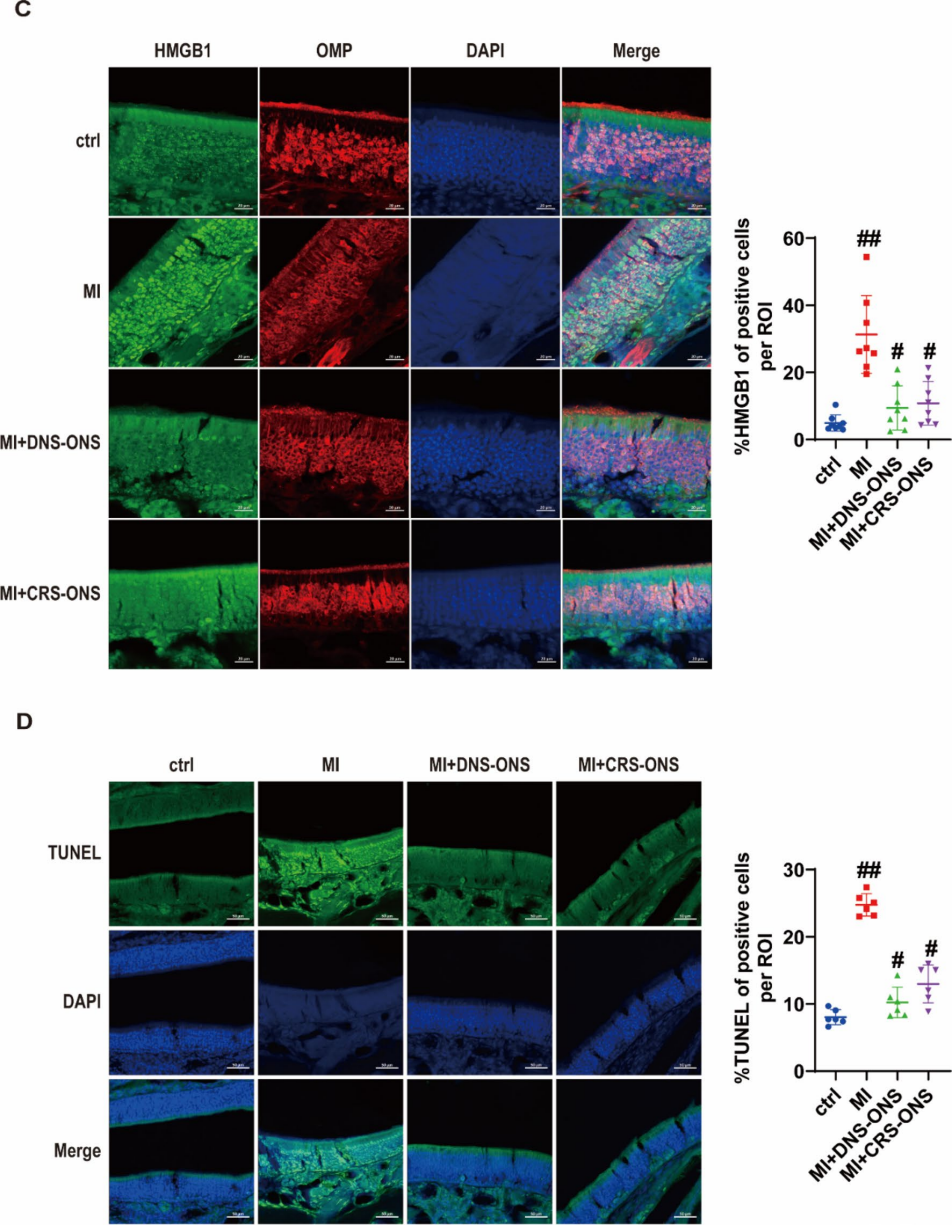

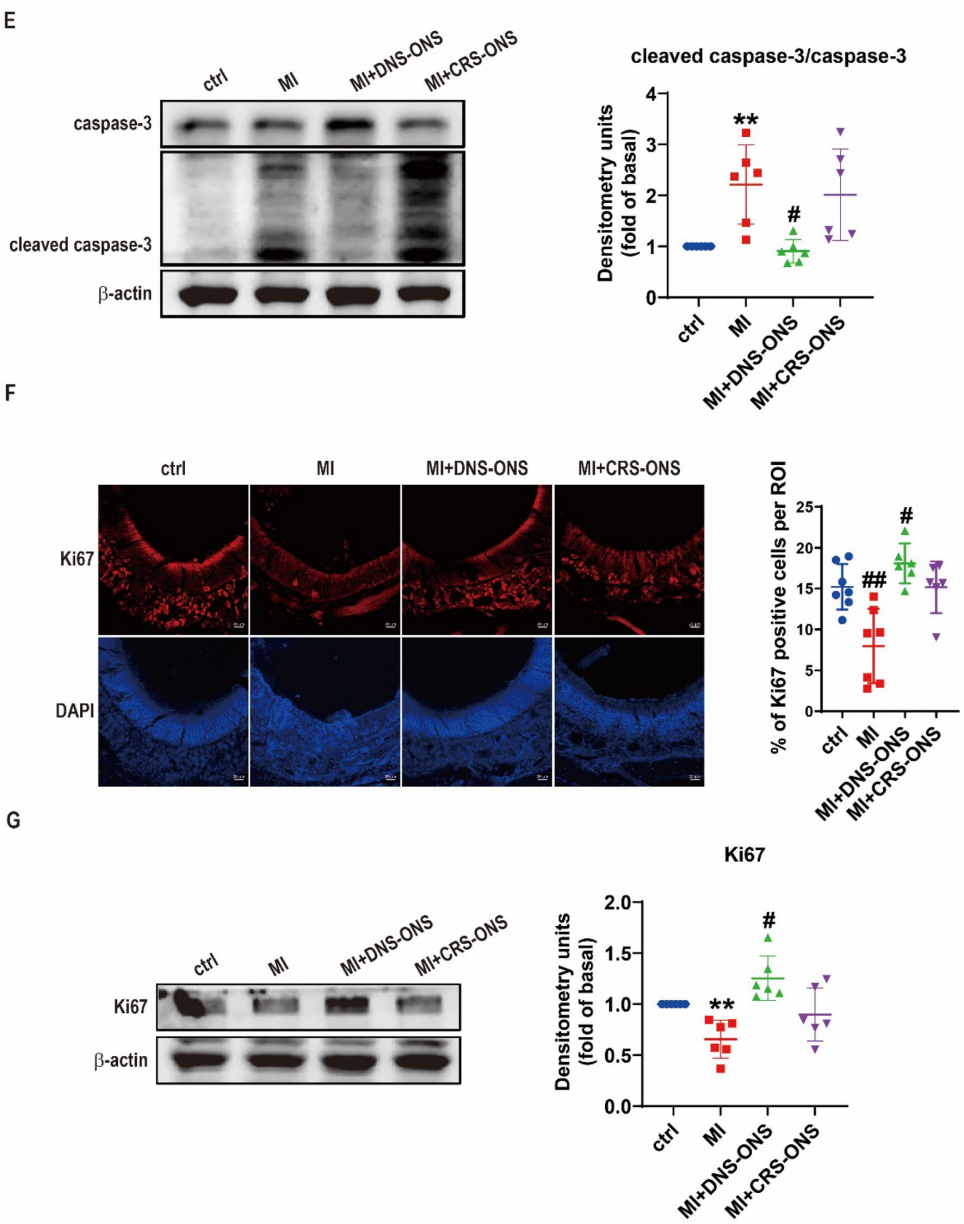
Impact of DNS- and CRS-ONS on cellular senescence, apoptosis, and proliferation in the OE of MI-Induced Mice. **A** Western blot analysis of cellular senescence markers in OE tissues, including γH2AX, p16, p21, and HMGB1. **B** Western blot analysis of pro-inflammatory cytokines IL-1β and IL-6 in OE tissues. **C** Immunofluorescence images showing HMGB1 and OMP expression in OE sections, with nuclei counterstained using DAPI. Scale bars, 20 µm. **D** Immunofluorescence images of TUNEL-positive cells in OE sections, indicating apoptosis, with nuclei counterstained using DAPI. Quantitative analysis of OMP in OE sections was performed with three samples per group, and two images per sample. Scale bars, 50 µm. **E** Western blot analysis of caspase-3 and cleaved caspase-3 levels in OE tissues. **F** Immunofluorescence images showing Ki67-positive cells in OE sections, indicating cell proliferation, with nuclei counterstained using DAPI. Scale bars, 20 µm. **G** Western blot analysis of Ki67 expression levels in OE tissues. Statistical analyses were performed using one-way ANOVA with Tukey’s post-hoc test. Data are presented as mean ± standard deviation. *P < 0.05 and #*P* < 0.01 for comparisons between MI and either DNS- or CRS-ONS treatment groups; **P < 0.05 and ##*P* < 0.01 for comparisons between control and MI groups (n = 5 per group)

To investigate the potential of ONS to ameliorate MI-induced olfactory dysfunction by enhancing the regenerative abilities of the OE through decreased inflammation, cellular senescence, and apoptosis while promoting proliferation. Previous studies have shown a link between increased neuroinflammation, cellular senescence, and reduced viability of hippocampal neural stem cells [32]. MI-induced mice showed increased protein levels of senescence markers (γH2AX, p16, p21, HMGB1) and SASP factors (IL-1β, IL-6) in OE tissue lysates. DNS-ONS treatment effectively reduced these levels, while CRS-ONS treatment only reduced γH2AX and IL-6 (Fig. 6A, B). MI-treated mice had elevated HMGB1-positive cells, significantly reduced by DNS-ONS, but only modestly by CRS-ONS (Fig. 6C). MI-induced mice showed increased TUNEL-positive cells (Fig. 6D) and cleaved caspase-3/caspase-3 ratio (Fig. 6E). DNS-ONS treatment reduced these levels significantly, while CRS-ONS had a modest effect. DNS-ONS treatment notably increased Ki67-positive cells, indicating enhanced proliferation, compared to controls and CRS-ONS (Fig. 6F). Ki67 protein levels were also elevated with DNS-ONS treatment (Fig. 6G). Our results suggest that DNS-derived ONS ameliorates olfactory dysfunction by reducing cellular senescence and apoptosis and enhancing cellular proliferation in the OE of MI-induced mice.

## Discussion

Olfactory stem cell transplantation has been shown to promote neuronal regeneration in animal models of neurological disorders such as spinal cord injury, stroke, and Parkinson’s disease [11, 33, 34]. A recent study demonstrated that treatment with murine adult olfactory stem cells rescues hyposmia in a mouse model [35]. However, a significant gap exists between stem cell research and its clinical application for treating olfactory dysfunction using autologous olfactory stem cells, especially considering patient-specific characteristics that may affect therapeutic efficacy. This study highlights the critical influence of cellular origin on the neurogenic potential of olfactory neurospheres.

In our culture system, ONS derived from the OE of patients contain both GBCs and HBCs, providing a distinct advantage as olfactory-specific stem cells compared to iPSC-derived neurospheres. While both DNS- and CRS-derived ONS exhibit neural stem cell properties (Nestin, SOX2, SOX1, and Pax6) and can be expanded efficiently, DNS-ONS demonstrated superior proliferative potential compared to CRS-ONS. Notably, SOX2 mRNA levels were significantly lower in CRS-ONS than in DNS-ONS. Given SOX2’s critical role in neural stem cell self-renewal, proliferation, and differentiation [36], its reduced expression in CRS-ONS likely explains their diminished proliferation rates, differentiation capacity, and neurotrophic factor production.

Differentiated cells from CRS-ONS also showed impaired maturation of olfactory sensory neurons (OSNs) and lower levels of neurotrophic factors such as BDNF and GDNF compared to DNS-ONS. Consequently, DNS-ONS treatment more effectively restored olfactory epithelium (OE) integrity and functionality in methimazole (MI)-induced hyposmic mice compared to CRS-ONS. This can be attributed to DNS-ONS’s ability to mitigate cellular senescence, reduce apoptosis, and promote cellular proliferation within the OE.

The OE has the capacity to produce multipotent neurospheres in vitro, which can differentiate into neurons and glia while maintaining the ability to self-renew [33]. In this study, OE-derived neurospheres expressed neural stem cell markers (SOX1, Pax6), GBC markers (ASCL1, Ngn1), and HBC markers (p63, KRT14). Notably, iPSC-derived neurospheres lacked ASCL1 and p63 expression, indicating that OE-derived ONS more closely resemble olfactory-specific stem cells. CRS-related impairments in stem cell functionality, as demonstrated by fewer proliferating cells and immature differentiation of OSNs in CRS patients [16], align with our findings of reduced proliferative and regenerative capabilities in CRS-ONS compared to DNS-ONS. Similarly, ONS derived from patients with nasal polyps and Alzheimer’s disease demonstrate a restricted capacity for both self-renewal and sphere-formation [37, 38]. Our observations support this, as CRS-ONS compared to DNS-ONS showed reduced proliferative capabilities and promoted immature differentiation of OSNs. This was evident from the decreased expression of Ki67, OMP, AC3, and Golf, and increased expression of GAP43 and TrkB in a differentiation medium.

Olfactory stem cells secrete neurotrophins (e.g., BDNF, GDNF, NGF, NT-3, NT-4, and CNTF) that promote nerve regeneration [39]. However, persistent inflammation in CRS patients inhibits olfactory stem cells from fully supporting neuron maturation and HBC differentiation [16]. In this study, CRS-ONS secreted significantly lower levels of BDNF and GDNF than DNS-ONS, potentially contributing to their reduced therapeutic efficacy.

Prior attempts have been made to transplant stem cells into the olfactory neuroepithelium. Human cord blood CD133-positive mesenchymal stem cells (MSCs) and adipose tissue-derived MSCs intravenously promote regeneration in the damaged olfactory region [40–42]. Bone marrow-MSCs, introduced into irradiated mice, can engraft into the OE, differentiate into olfactory neuronal cells, and restore olfactory functions by expressing NGF and BDNF [43, 44]. Transplantation of neural stem cells (NSCs) into an anosmic mouse model enhanced OMP expression in the OE and recovered olfactory functionality [2].

While MSC and NSC transplantation is promising, procuring MSCs from bone and adipose tissue and NSCs from the brain is invasive. In contrast, OE stem cells can be conveniently harvested from living patients through endoscopically-guided biopsy, making them attractive for cell-based therapies [45]. MSCs originate from mesenchymal embryonic tissue (mesoderm) [46], whereas olfactory epithelial cells arise from the neural crest [47], which can differentiate into neuroectodermal derivatives more effectively than MSCs. OE-derived stem cells show promise for neurogenesis in conditions like Parkinson’s disease [48] and spinal cord injury [49]. A recent study illustrated the therapeutic potential of murine olfactory stem cells in a mouse hyposmia model [35]. Our research demonstrates that human OE-derived ONS can effectively treat a methimazole-induced mouse hyposmia model. This treatment fostered the development of olfactory neurons, indicated by elevated OMP and AC3 levels in the MI-damaged OE. Additionally, there was an enhancement in olfactory epithelial integrity and thickness, coupled with noticeable recovery at the behavioral level. DNS-derived ONS exhibited superior restorative efficacy compared to CRS-ONS. This aligns with our earlier in vitro study findings, suggesting that CRS-ONS’s differentiation into immature OSNs and reduced BDNF and GDNF production might cause its diminished in vivo therapeutic efficacy compared to DNS-ONS.

Cellular senescence, characterized by a pro-inflammatory phenotype, hinders tissue regeneration and has been linked to a diminished structural response in the OE [50]. Mitigating apoptosis in OSNs is crucial for restoring both OE thickness and olfactory function [51]. Our study observed the generation of new cells in MI-damaged OE post-ONS treatment, evidenced by the surge in Ki67-positive cells in MI-induced mice following DNS-ONS treatment. We hypothesized that ONS treatment could combat cellular senescence and apoptosis while fostering cell generation. Supporting this, we detected heightened levels of markers for cell senescence—p16, p21, γH2AX, HMGB1, and pro-inflammatory cytokines IL-1β and IL-6—in the OE of MI-induced mice. These markers were notably diminished post-DNS-ONS treatment. Similarly, the prevalence of TUNEL-positive cells and the expression of caspase-3 were significantly lower in the DNS-ONS-treated groups than in the untreated MI-induced mice. In essence, ONS treatment bolsters the health of the OSNs in MI-damaged OE by mitigating cellular senescence and apoptosis while stimulating new cell generation. It is worth noting that while CRS-ONS treatment led to a reduction in cellular senescence, apoptosis, and proliferation in the MI-damaged OE, its effects were more subdued compared to DNS-ONS.

Our study showed that ONS derived from patients with DNS and CRS have varying abilities in proliferation and differentiation into OSNs in vitro, correlating with their therapeutic efficacy in the hyposmic mouse model. Notably, CRS-ONS demonstrated limited potential for self-renewal and differentiation into mature OSNs, highlighting potential hurdles for therapeutic applications. Understanding the quality of the stem cell source is crucial for its application in regenerative medicine, especially in autologous stem cell transplantation where a patient’s disease history must be considered. Future research should identify specific factors within CRS-ONS that hinder proliferation and the differentiation and maturation of OSNs. Addressing these factors could pave the way for tailored therapeutic strategies to enhance olfactory function recovery.

## Conclusions

Selecting appropriate stem cell sources is crucial for effectively treating olfactory dysfunctions. Among the sources studied, ONS derived from patients with a DNS demonstrate significantly greater potential in regenerating the olfactory epithelium and restoring olfactory function compared to those derived from CRS. The superior self-renewal capacity, enhanced production of neurotrophic factors, and greater efficacy in reducing cellular senescence and apoptosis position DNS-derived ONS as a more promising candidate for therapeutic applications in olfactory dysfunction.

## Supplementary Information

Additional file 1.

Additional file 2.

Additional file 3.

Additional file 4.

## Data Availability

The datasets used during the current study are available from the corresponding authors on reasonable request.

## Abbreviations

CRS: Chronic rhinosinusitis
DNS: Deviated nasal septum
OSNs: Olfactory sensory neurons
ONS: Olfactory neurospheres
OE: Olfactory epithelium
MI: Methimazole
OMP: Olfactory marker protein
ADCY3: Adenylyl cyclase 3
