# Supplementary material for: Differential efficacy of olfactory neurospheres from deviated nasal septum and chronic rhinosinusitis patients in regenerating olfactory epithelium
Source: Stem Cell Res Ther. 2025 Apr 5;16:166. doi: 10.1186/s13287-025-04270-0 (PMC11972463; doi:10.1186/s13287-025-04270-0)
Supplement: Supplementary file 1 — Additional file 1. [file 13287_2025_4270_MOESM1_ESM.docx]

**Additional file 1: Table S1.** Antibodies used in the study.

| **Antibodies** | **Company** | **Catalog No.** | **Application (dilution)** |
| --- | --- | --- | --- |
| Ki67 | Abcam | Ab15580 | IF (1:100) |
| BrdU | Santa Cruz | sc-32323 | IF (1:100) |
| Nestin | BioLegend | 841901 | IF (1:100) |
| Pax6 | EMD Millipore | MAB5552 | IF (1:100) |
| p63 | Abcam | ab124762 | IF (1:100),  FCM (1:100),  WB (1:500) |
| ASCL1 | Santa Cruz | sc-374104 | IF (1:100),  FCM (1:100), WB (1:500) |
| ICAM-1 | Santa Cruz | sc-8439 | WB (1:1000) |
| SOX-2 | EMD Millipore | MAB4343 | WB (1:500) |
| MAP2 | Santa Cruz | sc-74421 | IF (1:100) |
| OMP | Santa Cruz | sc-365818 | IF (1:100),  IHC (1:100),  FCM (1:100),  WB (1:500) |
| AC3 | Novus Biologicals | NBP1-92683 | IF (1:100),  IHC (1:100),  FCM (1:100),  WB (1:500) |
| Golf | Santa Cruz | sc-55545 | FCM (1:100),  WB (1:500) |
| TrkB | Abcam | ab134155 | WB (1:500) |
| GAP43 | GeneTex | GTX127937 | WB (1:1000) |
| BDNF | Abcam | ab203573 | WB (1:500) |
| GDNF | ABclonal | A14639 | WB (1:1000) |
| γH2AX | GeneTex | GTX127340 | WB (1:500) |
| p16 | GeneTex | GTX03119 | WB (1:500) |
| P21 | ABclonal | A19094 | WB (1:500) |
| HMGB1 | Cell Signaling Technology | #3935 | WB (1:500),  IHC (1:100) |
| IL-1β | Cell Signaling Technology | #12242 | WB (1:500) |
| IL-6 | Santa Cruz | sc-57315 | WB (1:500) |
| Caspase-3 | Novus Biologicals | 31A1067 | WB (1:500) |
| β-actin | Abcam | ab8227 | WB (1:1000) |
| Lamin A | Santa Cruz | sc-376248 | WB (1:1000) |
| TUNEL | Invitrogen | A23210 | IHC |
| DyLight™ 549 Anti-Mouse IgG Antibody, | Vector Lab | DI-2549 | IF, IHC |
| DyLight™ 488 Anti-Rabbit IgG, | Vector Lab | DI-1088 | IF, IHC |
| DAPI | Vector Lab | H-1800 | IF, IHC |
| Goat Anti-Mouse IgG | Jackson ImmunoReasearch Lab | 115-035-003 | WB |
| Goat-Anti-Rabbit IgG | Jackson ImmunoReasearch Lab | 111-035-003 | WB |

FCM: Flow cytometry. IF: immunofluorescence. IHC: immunohistochemistry. WB: western blot.

**Additional file 1: Table S2.** Sequence of primers used for RT-PCR studies.

| **Gene** | **Forward primer (5'-3')** | **Reverse primer (5'-3')** |
| --- | --- | --- |
| SOX1 | GAAGCCCAGATGGAAATACG | GGACAAGGAAGGGTGTTGAG |
| SOX2 | TTGCTGCCTCTTTAAGACTAGGA | TAAGCCTGGGGCTCAAACT |
| Pax6 | TCCGTTGGAACTGATGGAGT | GTTGGTATCCGGGGACTTC |
| Nestin | TCAAGATGTCCCTCAGCCTGGA | AAGCTGAGGGAAGTCTTGGAGC |
| ASCL1 | TCTCATCCTACTCGTCGGACGA | CTGCTTCCAAAGTCCATTCGCAC |
| Ngn1 | GCCTCCGAAGACTTCACCTACC | GGAAAGTAACAGTGTCTACAAAGG |
| p63 | CAGGAAGACAGAGTGTGCTGG T | AATTGGACGGCGGTTCATCCCT |
| KRT14 | CCATTGAGGACCTGAGGAAC | CAATCTGCAGAAGGACATTGG |
| OMP | CAG CGG AGTCTGTGTACCG | TCCAGCGCTCGAACTGTAG |
| AC3 | GCTGGAGGTGAAGATGAACC | CTTGGGCAGGATGGAAAG |
| BDNF | CATCCGAGGACAAGGTGGCTTG | GCCGAACTTTCTGGTCCTCATC |
| GDNF | TCATTCGTGGAGCACAATTT | CAAGGTTCTCTGAATGGGACA |
| GAPDH | GTCTCCTCTGACTTCAACAGCG | ACCACCCTGTTGCTGTAGCCAA |
