# Supplementary material for: Differential efficacy of olfactory neurospheres from deviated nasal septum and chronic rhinosinusitis patients in regenerating olfactory epithelium
Source: Stem Cell Res Ther. 2025 Apr 5;16:166. doi: 10.1186/s13287-025-04270-0 (PMC11972463; doi:10.1186/s13287-025-04270-0)
Supplement: Supplementary file 3 — Additional file 3. [file 13287_2025_4270_MOESM3_ESM.docx]

**Supplementary Figure 1.**

**"Generation and differentiation of neural stem cells derived from iPSCs into neurons**

(A) A schematic representation illustrates the experimental paradigm for differentiating neural stem cells (NSCs) from induced pluripotent stem cells (iPSCs) over a total period of 18 days. Floating embryoid bodies (EBs) were generated from iPSCs, induced to form neural rosettes, and subsequently dissociated into NSCs. Representative images show the different stages of cell culture. (B) Representative phase-contrast images depict neural rosettes derived from iPSCs, with scale bars set at 50 μm. Fluorescent immunostaining images show iPSC-derived rosettes expressing the neural progenitor markers PAX6 and SOX1. Scale bars, 75 μm. (C) Phase-contrast images illustrate the morphology of NSCs in early (P3) and late passages (P30), demonstrating consistent morphology during long-term culture. Fluorescent immunostaining images show early- and late-passage NSCs expressing NESTIN and SOX1, with nuclei counterstained using DAPI. Scale bars, 50 μm. (D) RT-qPCR analysis was performed to assess the expression of neural progenitor genes (SOX1, PAX6, and NESTIN) and pluripotency genes (NANOG and OCT4) in early- and late-passage NSCs. The Y-axis represents gene expression levels relative to undifferentiated iPSCs, normalized to GAPDH. The data, derived from three independent differentiations, are represented as mean ± SEM. N.S. indicates non-significant differences. (E) Using a feeder-free, chemically defined in vitro differentiation protocol, human iPSCiNSCs were differentiated into neurons. Immunostaining revealed the typical morphology of neurons, with differentiated neurons expressing the neuronal markers MAP2 and Neuron D1.
