# Supplementary material for: Differential efficacy of olfactory neurospheres from deviated nasal septum and chronic rhinosinusitis patients in regenerating olfactory epithelium
Source: Stem Cell Res Ther. 2025 Apr 5;16:166. doi: 10.1186/s13287-025-04270-0 (PMC11972463; doi:10.1186/s13287-025-04270-0)
Supplement: Supplementary file 4 — Additional file 4. [file 13287_2025_4270_MOESM4_ESM.pdf]

Figure 2C

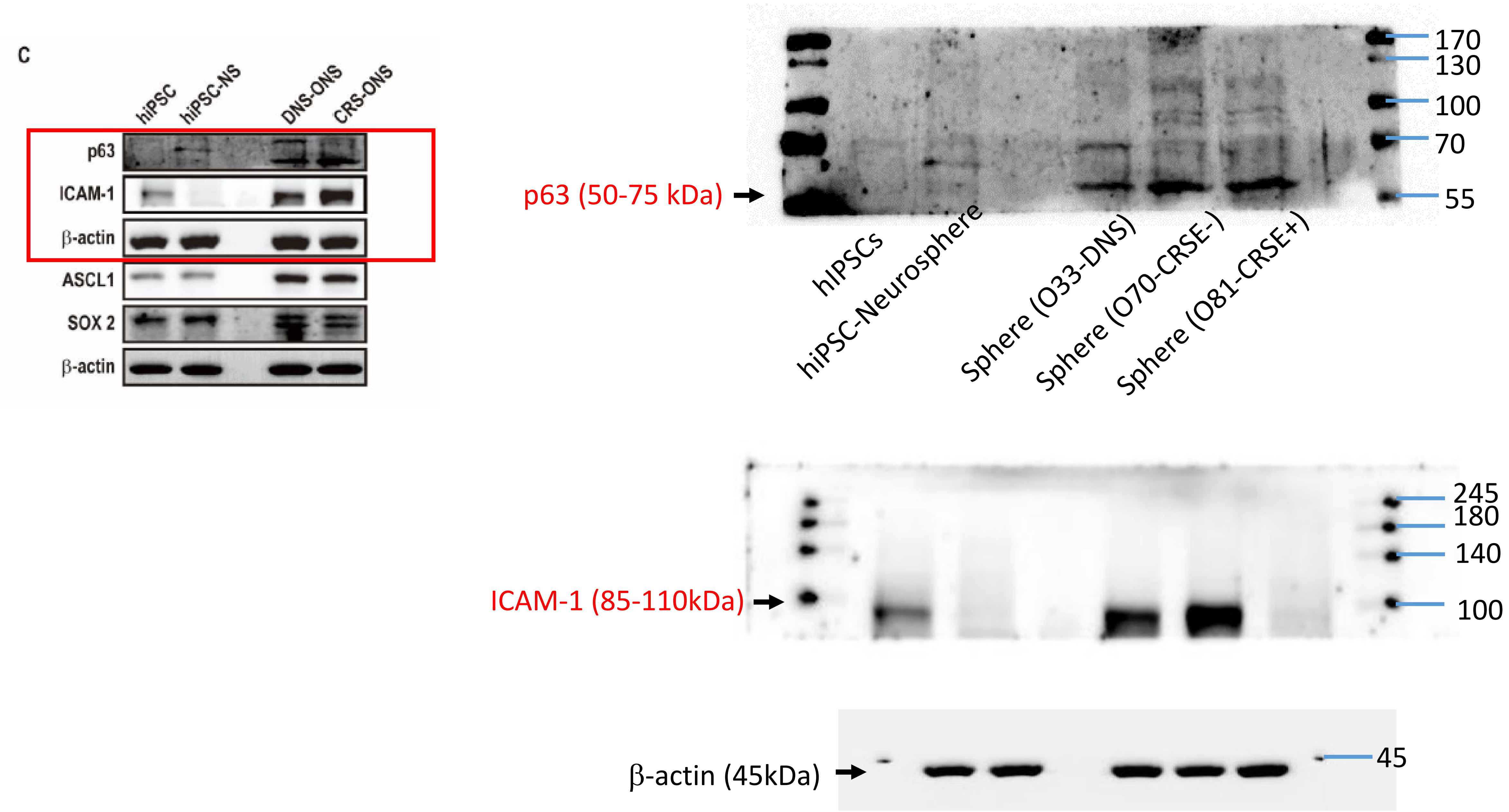
C
hiPSC
hiPSC-NS
DNS-ONS
CRS-ONS
p63
ICAM-1
β-actin
ASCL1
SOX 2
β-actin
p63 (50-75 kDa)
170
130
100
70
55
hIPSCs
hiPSC-Neurosphere
Sphere (O33-DNS)
Sphere (O70-CRSE-)
Sphere (O81-CRSE+)
ICAM-1 (85-110kDa)
245
180
140
100
β-actin (45kDa)
45

Figure 2C

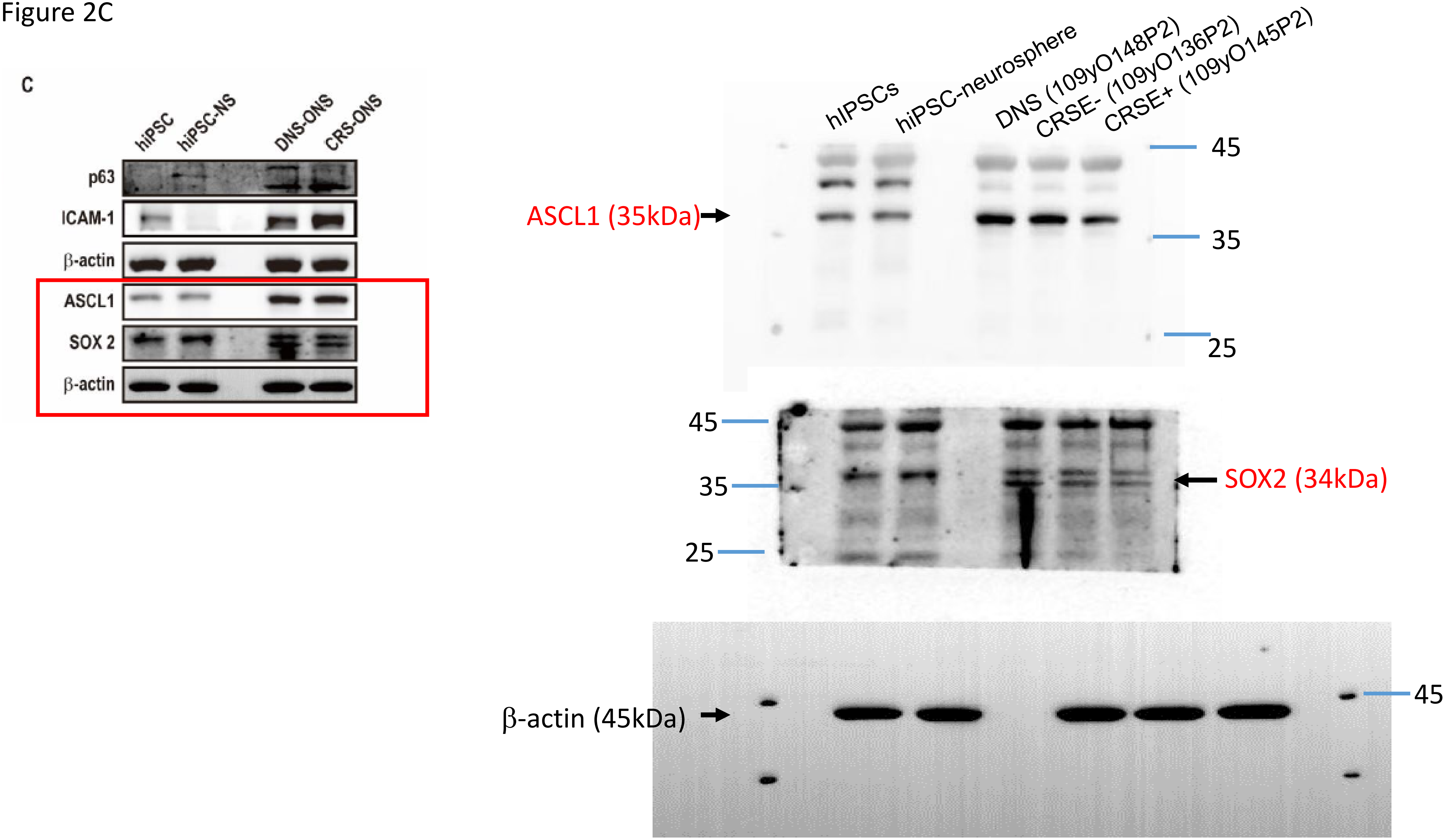

C
hiPSC
hiPSC-NS
DNS-ONS
CRS-ONS
p63
ICAM-1
β-actin
ASCL1
SOX 2
β-actin
hIPSCs
hiPSC-neurosphere
DNS (109yO148P2)
CRSE- (109yO136P2)
CRSE+ (109yO145P2)
45
35
25
ASCL1 (35kDa)
45
35
25
SOX2 (34kDa)
β-actin (45kDa)
45

Figure 3C

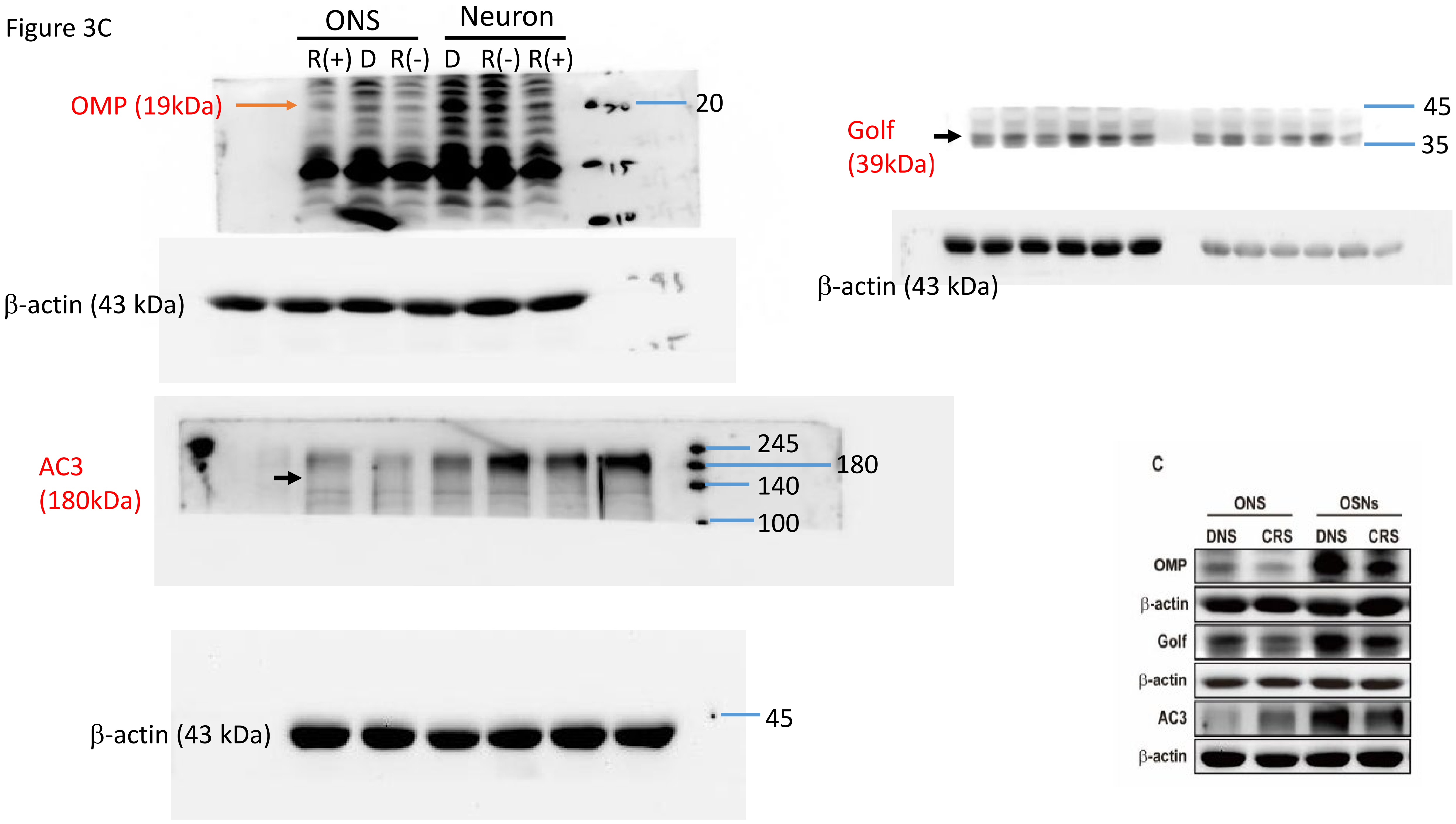

ONS
Neuron
R(+) D R(-) D R(-) R(+)
OMP (19kDa)
20
β-actin (43 kDa)
Golf
(39kDa)
45
35
β-actin (43 kDa)
AC3
(180kDa)
245
180
140
100
β-actin (43 kDa)
45
C
ONS
OSNs
DNS CRS DNS CRS
OMP
β-actin
Golf
β-actin
AC3
β-actin

Figure 4A

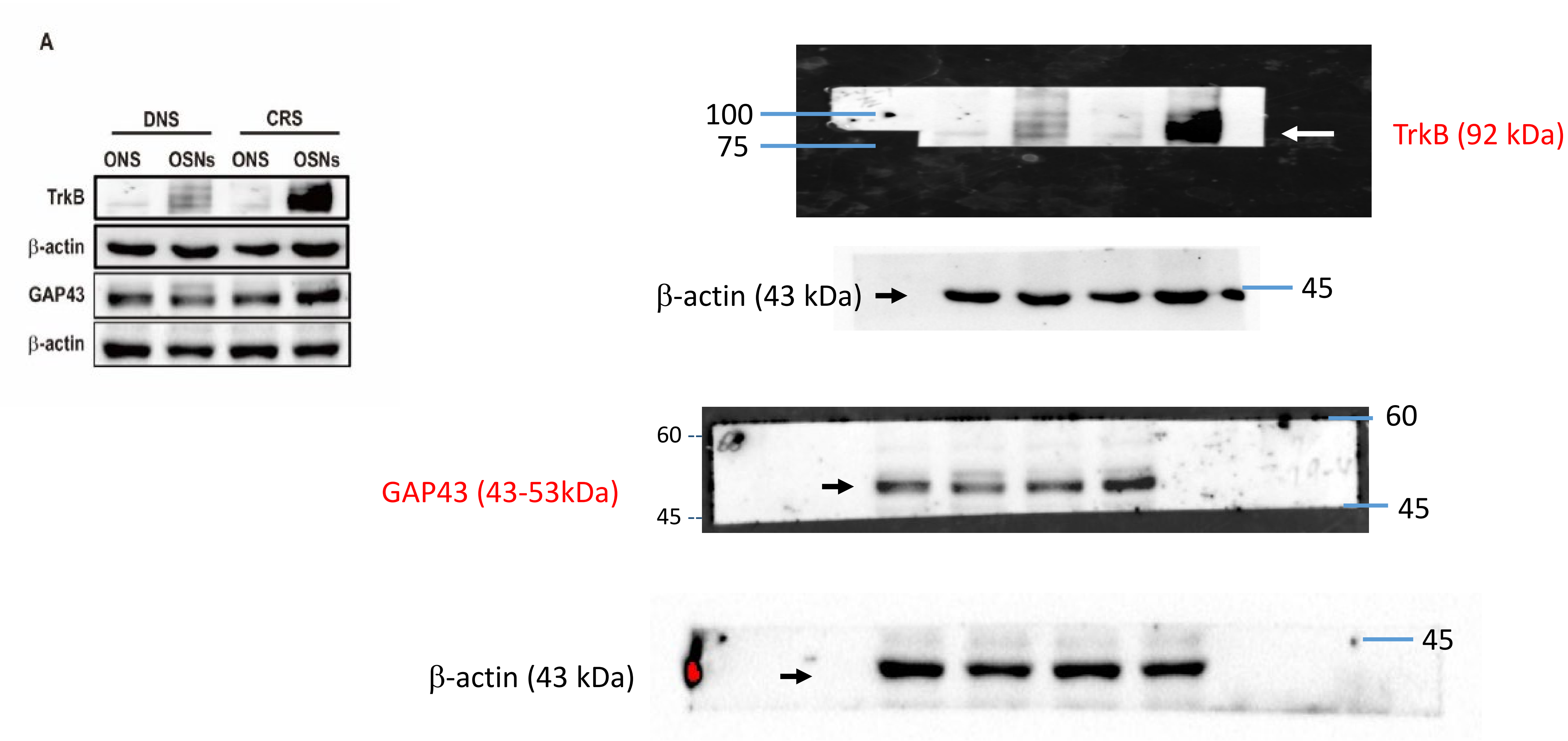
A
DNS
CRS
ONS
OSNs
ONS
OSNs
TrkB
β-actin
GAP43
β-actin
100
75
TrkB (92 kDa)
β-actin (43 kDa)
45
60
GAP43 (43-53kDa)
60
45
45
β-actin (43 kDa)
45

Figure 4C

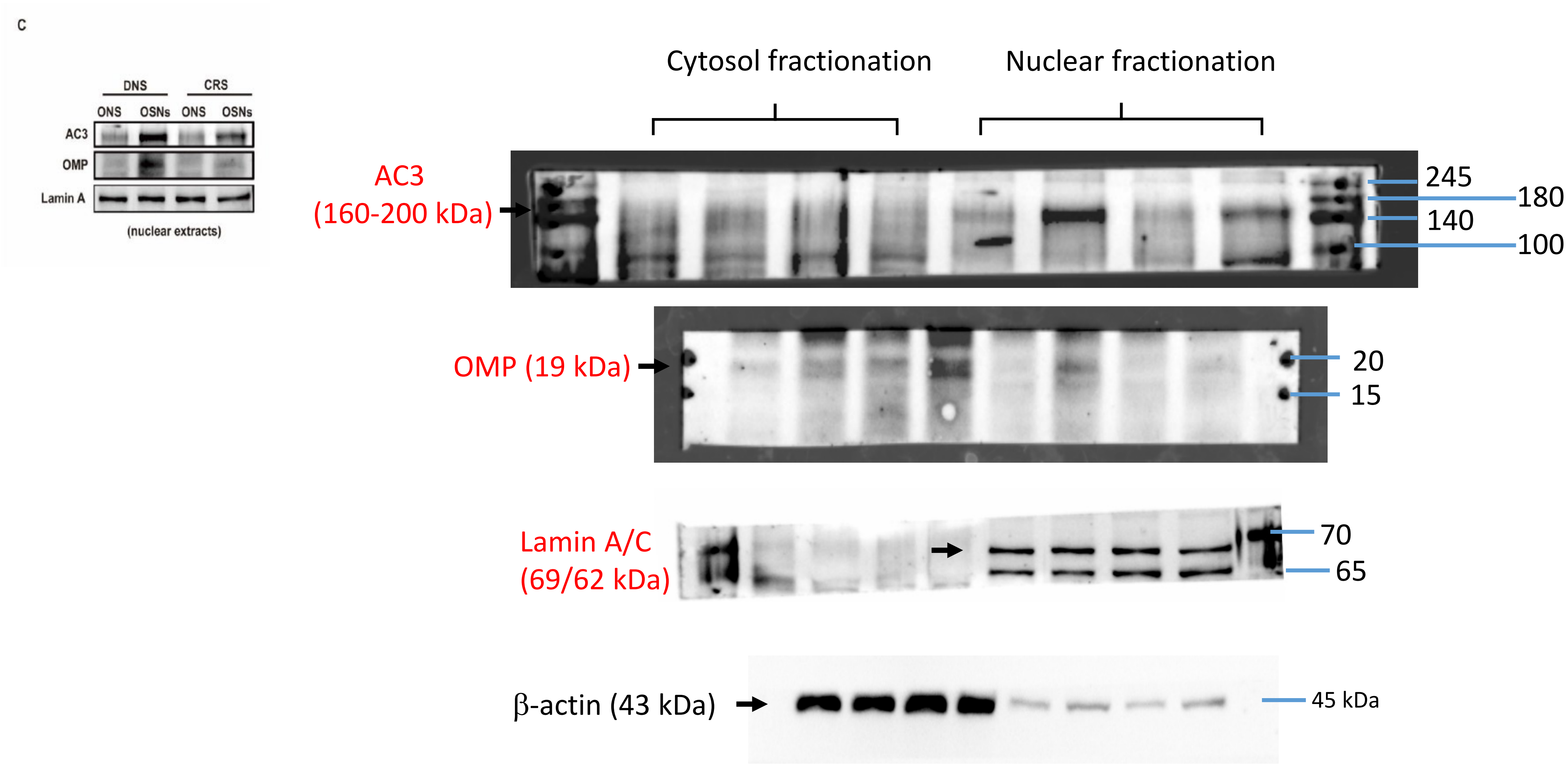

C
DNS
CRS
ONS
OSNs
ONS
OSNs
AC3
OMP
Lamin A
(nuclear extracts)
Cytosol fractionation
Nuclear fractionation
AC3
(160-200 kDa)
245
180
140
100
OMP (19 kDa)
20
15
Lamin A/C
(69/62 kDa)
70
65
β-actin (43 kDa)
45 kDa

Figure 4E

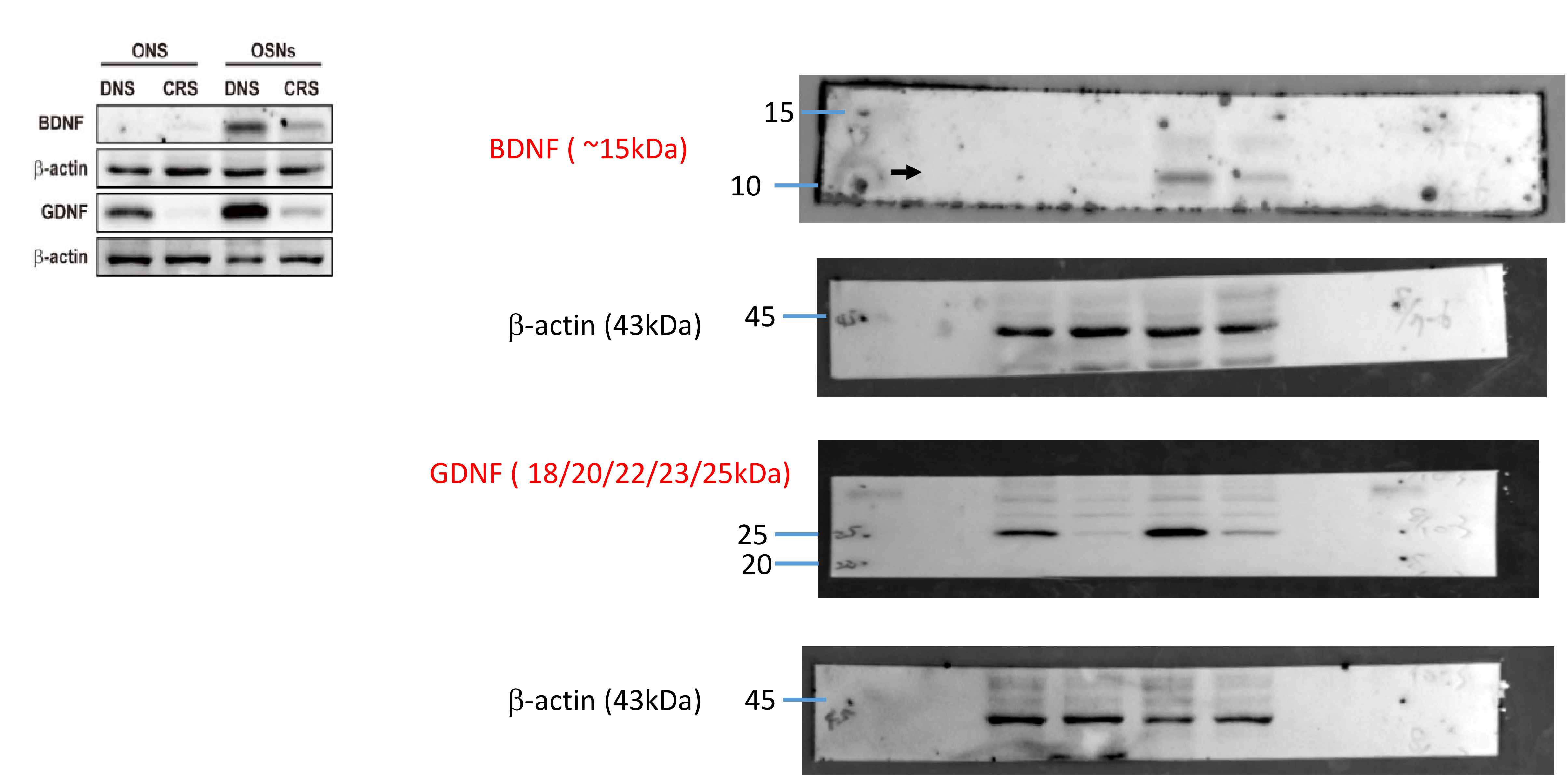

ONS
OSNs
DNS
CRS
DNS
CRS
BDNF
β-actin
GDNF
β-actin
BDNF (~15kDa)
15
10
β-actin (43kDa)
45
GDNF (18/20/22/23/25kDa)
25
20
β-actin (43kDa)
45

Figure 5D

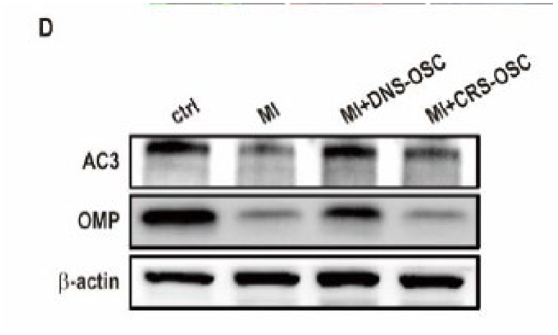
D
ctrl
MI
MI+DNS-OSC
MI+CRS-OSC
AC3
OMP
β-actin

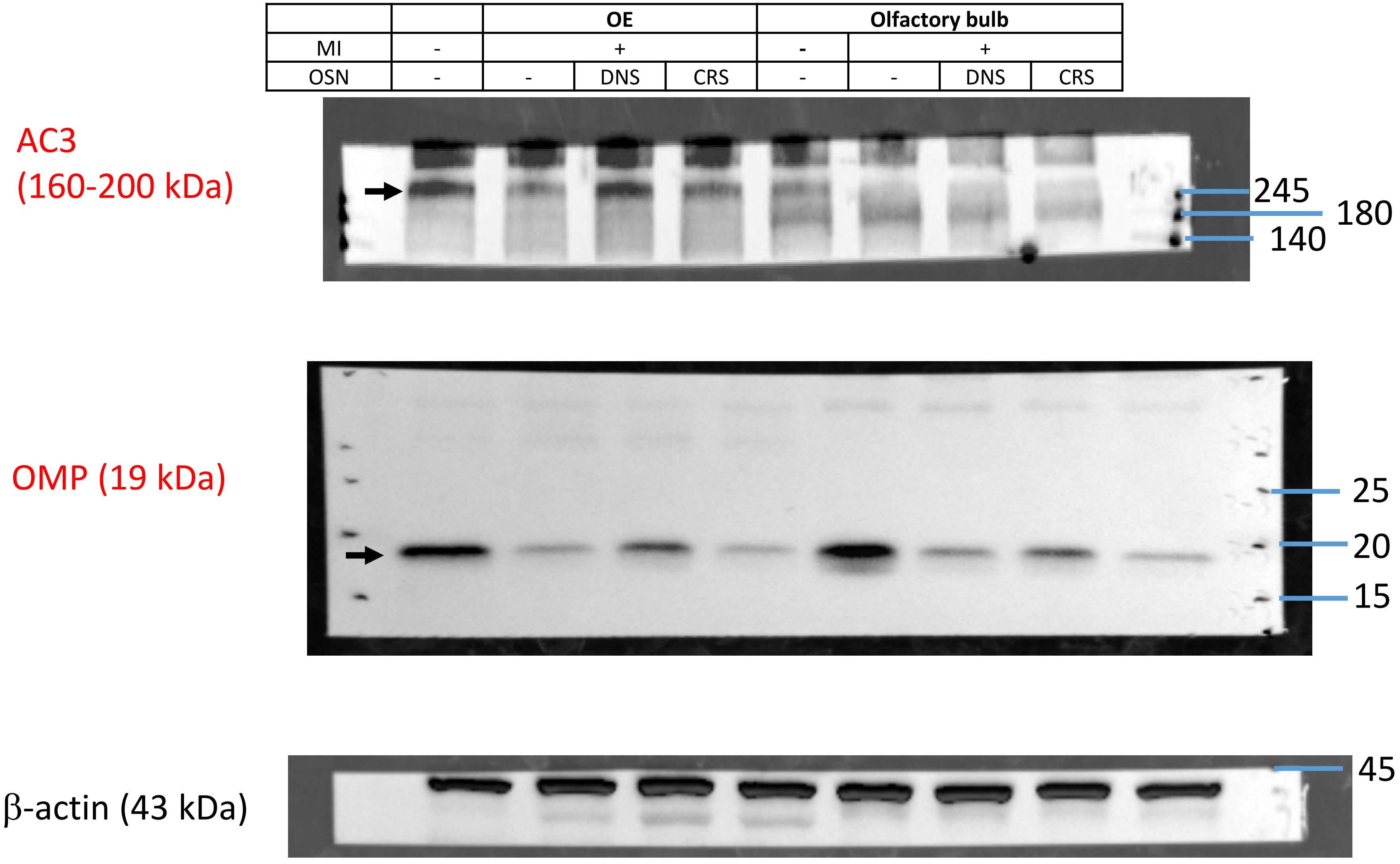
OE
Olfactory bulb
MI - + - +
OSN - - DNS CRS - - DNS CRS
AC3
(160-200 kDa)
245
180
140
OMP (19 kDa)
25
20
15
β-actin (43 kDa)
45

Figure 6A

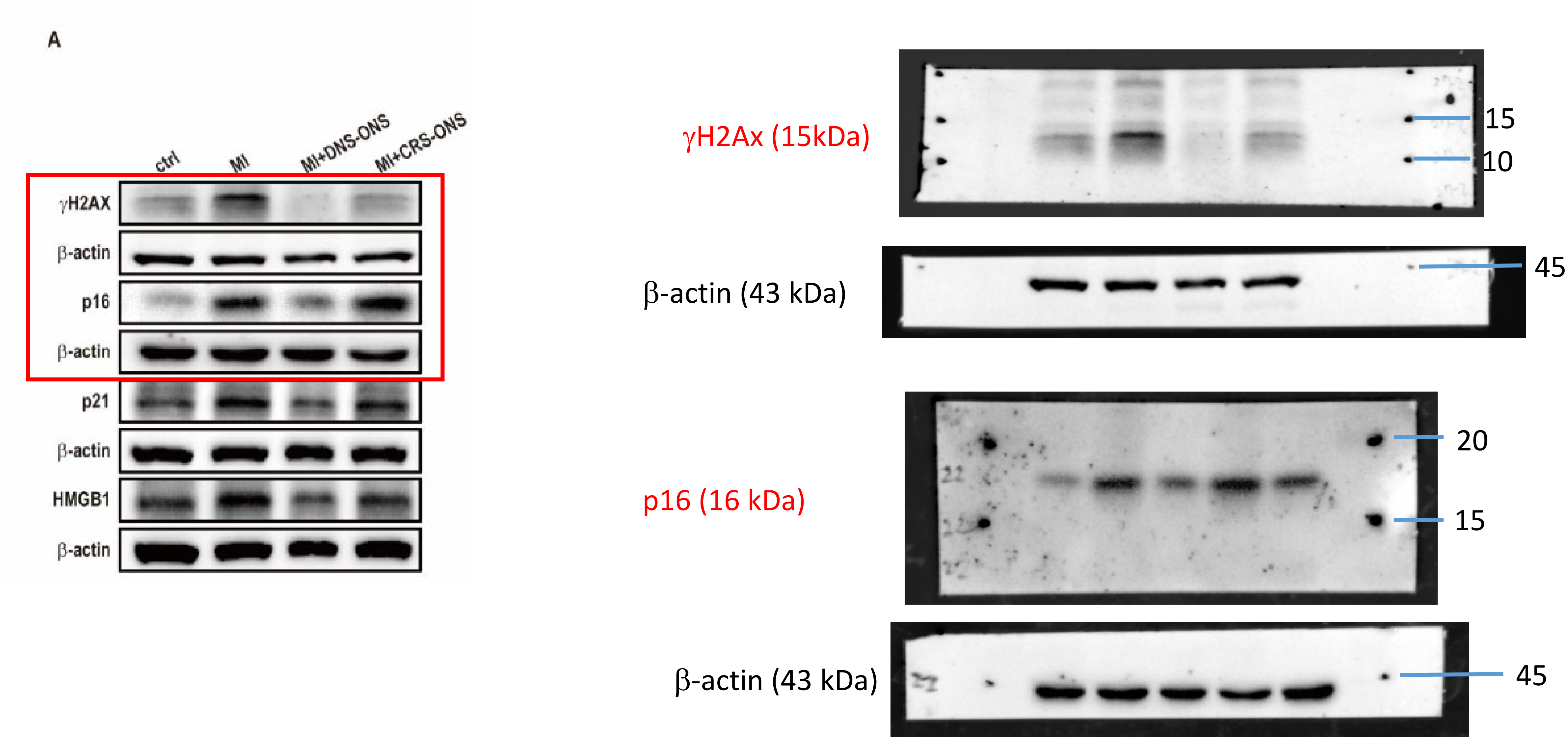

A
ctrl
MI
MI+DNS-ONS
MI+CRS-ONS
γH2AX
β-actin
p16
β-actin
p21
β-actin
HMGB1
β-actin
γH2Ax (15kDa)
15
10
β-actin (43 kDa)
45
p16 (16 kDa)
20
15
β-actin (43 kDa)
45

Figure 6A

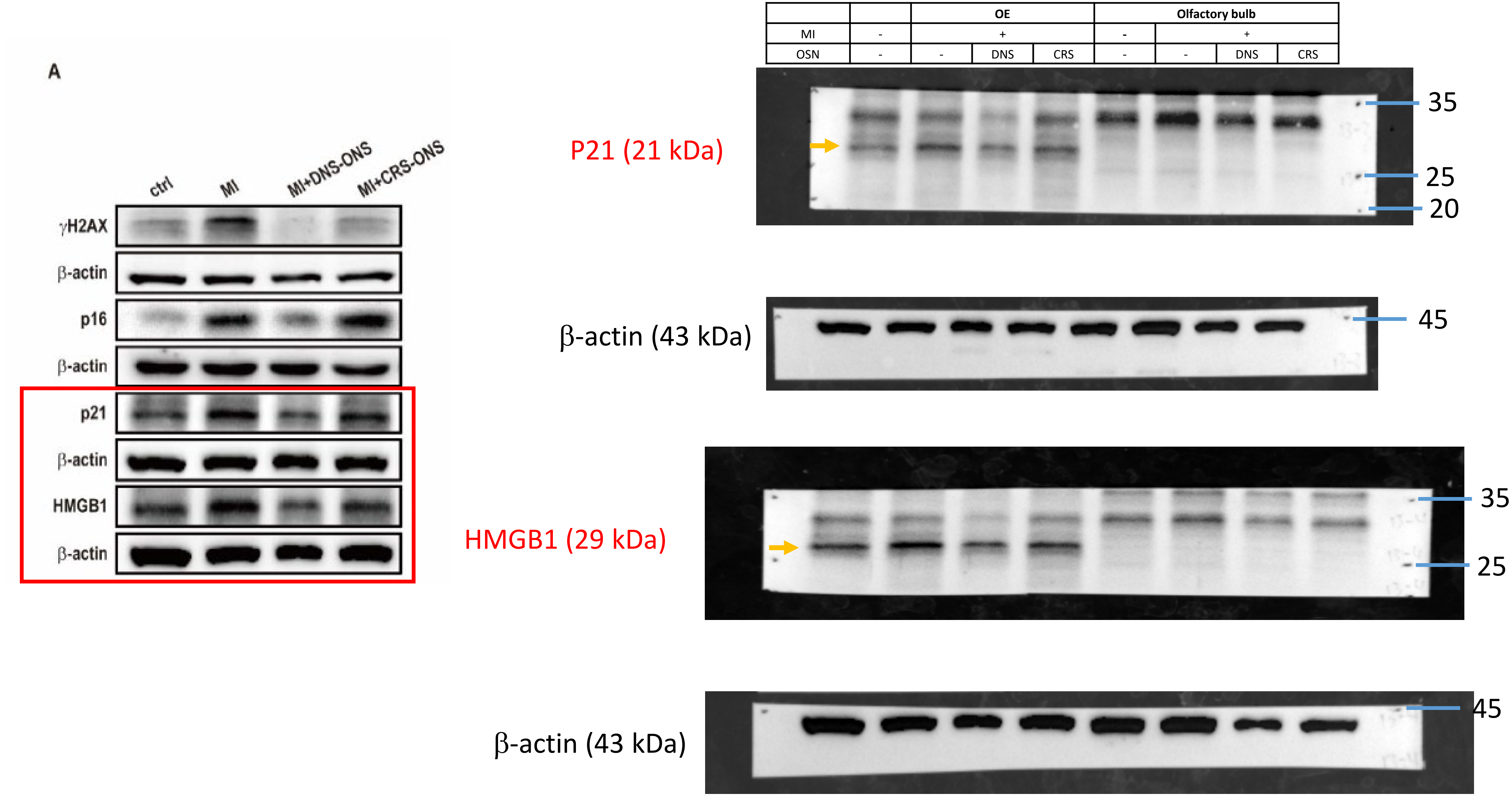
A
ctrl
MI
MI+DNS-ONS
MI+CRS-ONS
γH2AX
β-actin
p16
β-actin
p21
β-actin
HMGB1
β-actin
OE
Olfactory bulb
MI
-
+
-
+
OSN
-
-
DNS
CRS
-
-
DNS
CRS
P21 (21 kDa)
35
25
20
β-actin (43 kDa)
45
HMGB1 (29 kDa)
35
25
β-actin (43 kDa)
45

Figure 6B

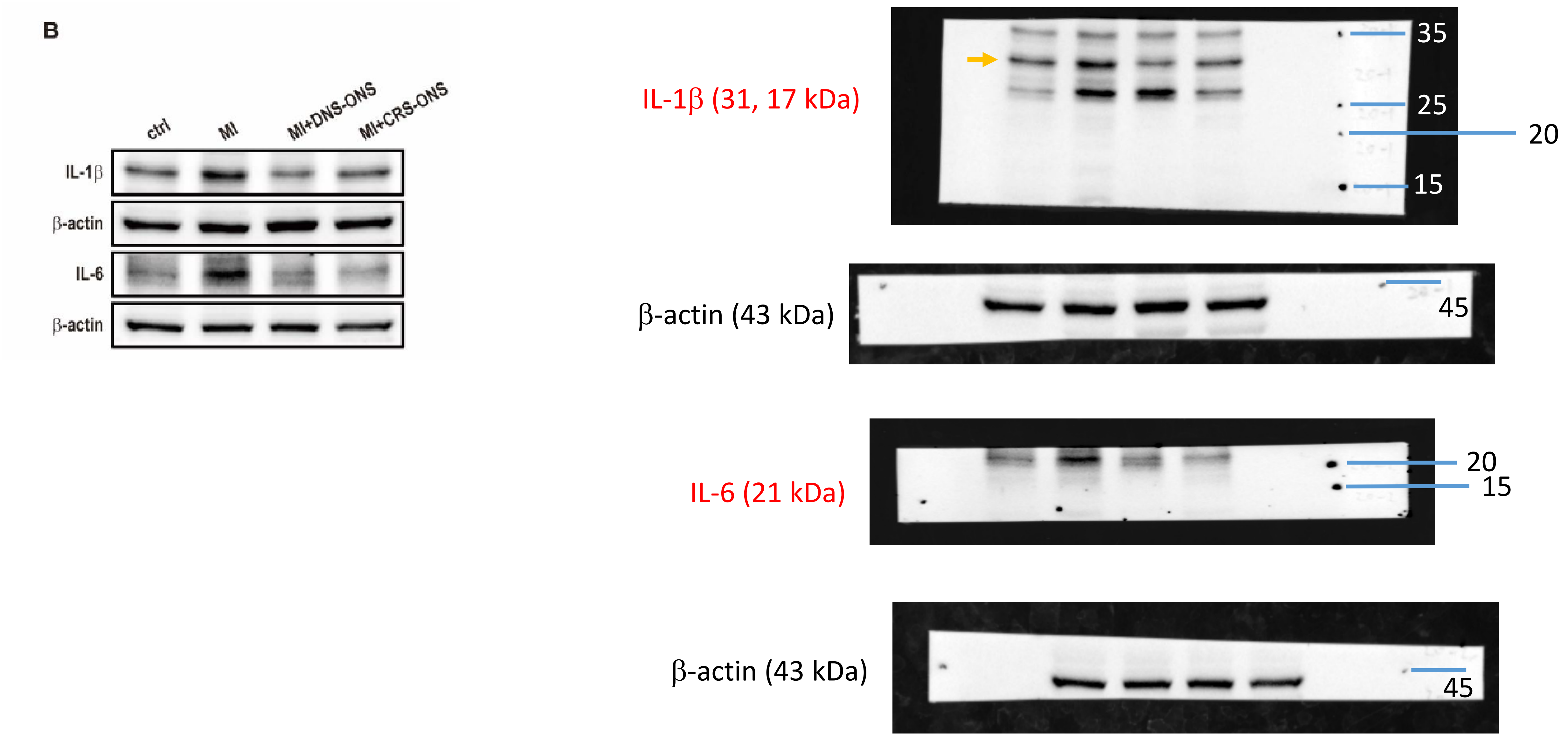
B
ctrl
MI
MI+DNS-ONS
MI+CRS-ONS
IL-1β
β-actin
IL-6
β-actin
IL-1β (31, 17 kDa)
35
25
20
15
β-actin (43 kDa)
45
IL-6 (21 kDa)
20
15
β-actin (43 kDa)
45

Figure 6E

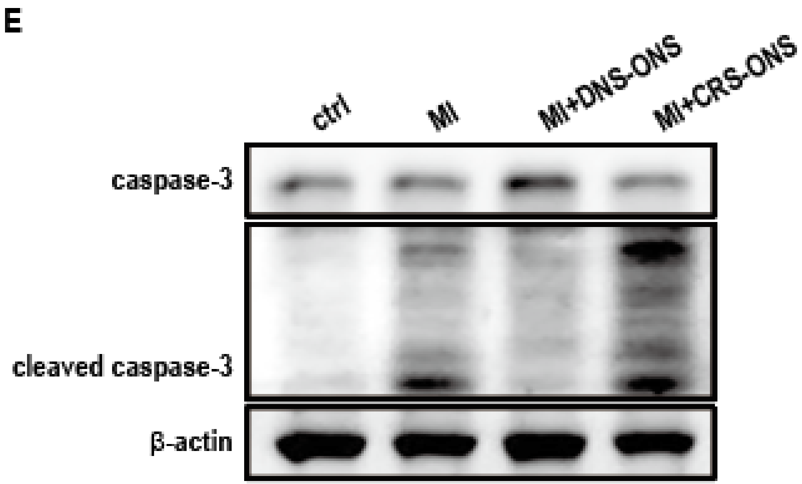

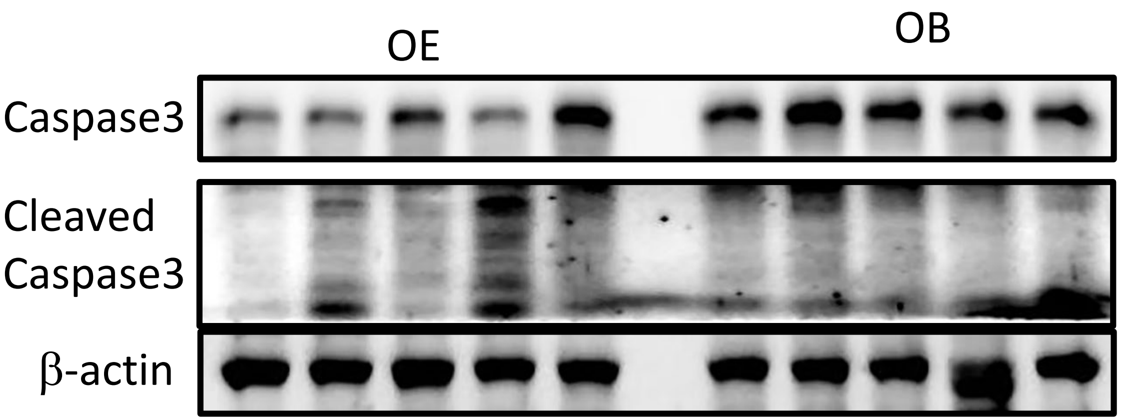

MI	-	+					-	+			
OSC	-	-	DNS	CRS E-	CRS E+		-	-	DNS	CRS E-	CRS E+
8th(Lasco)	B2	D3	A1		C2		B2	D3	A1		C2
9th(國動)				C3						C3	

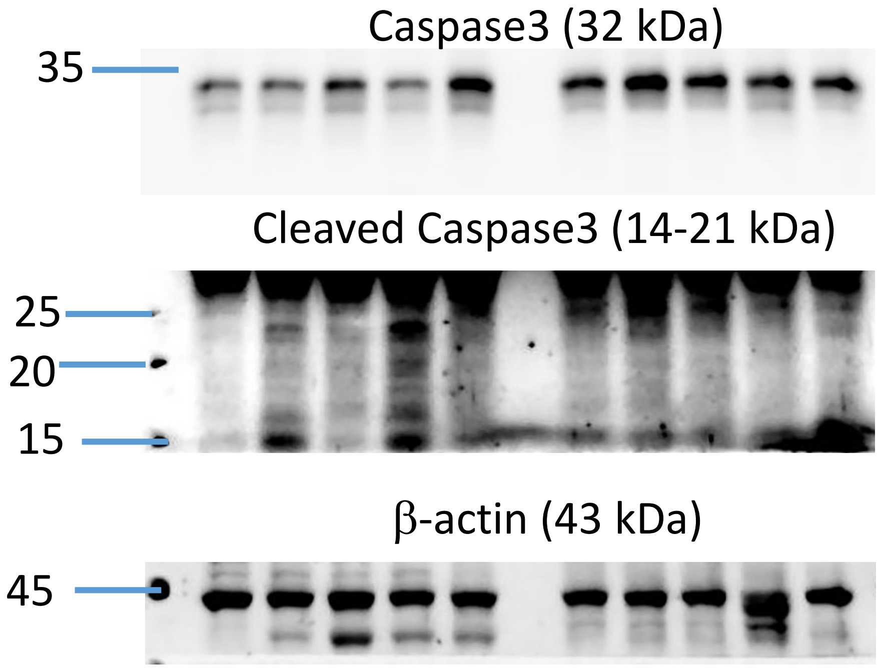

Figure 6G

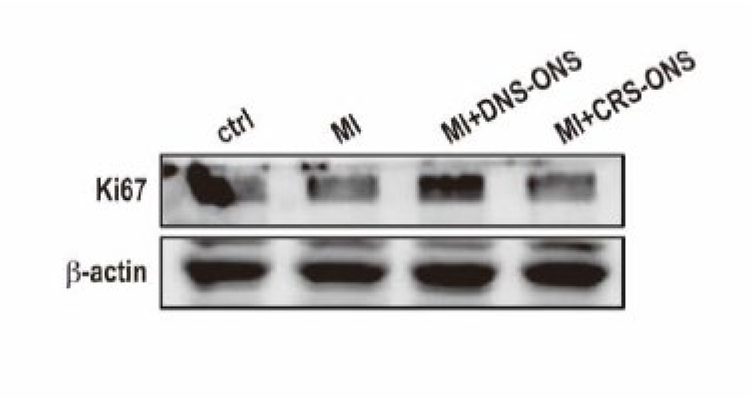
ctrl
MI
MI+DNS-ONS
MI+CRS-ONS
Ki67
β-actin

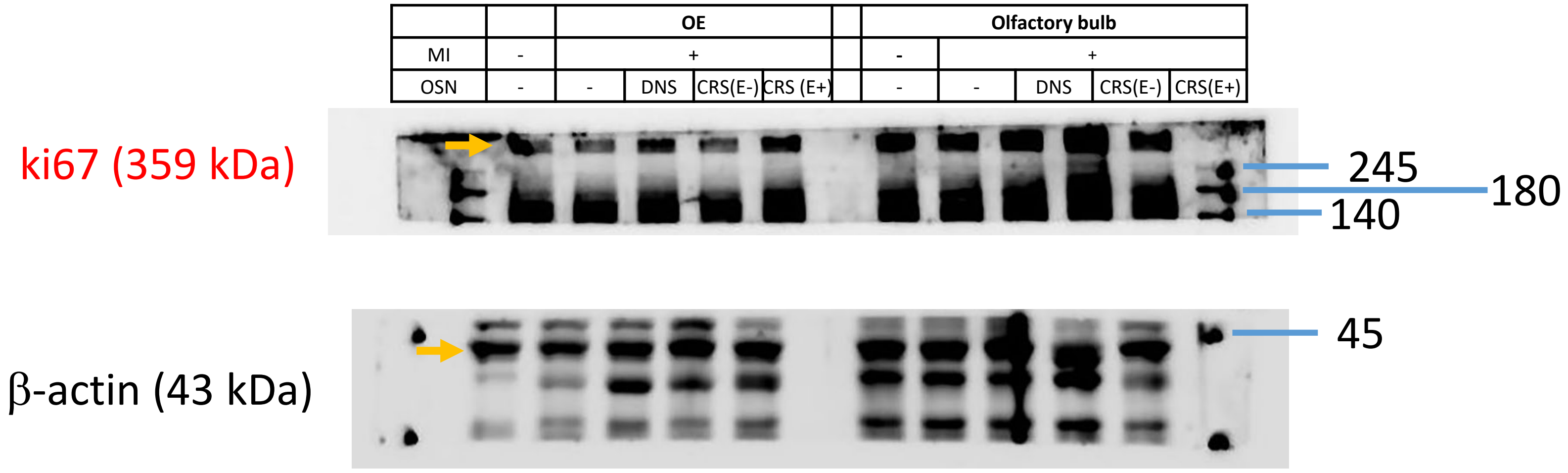
OE
Olfactory bulb
MI
-
+
-
+
OSN
-
-
DNS
CRS(E-)
CRS (E+)
-
-
DNS
CRS(E-)
CRS(E+)
ki67 (359 kDa)
245
180
140
β-actin (43 kDa)
45
